# Supplementary figures and images for: Transcriptome analysis of Curcuma wenyujin from Haikou and Wenzhou, and a comparison of the main constituents and related genes of Rhizoma Curcumae
Source: PLoS One. 2020 Nov 30;15(11):e0242776. doi: 10.1371/journal.pone.0242776 (PMC7703983; doi:10.1371/journal.pone.0242776)

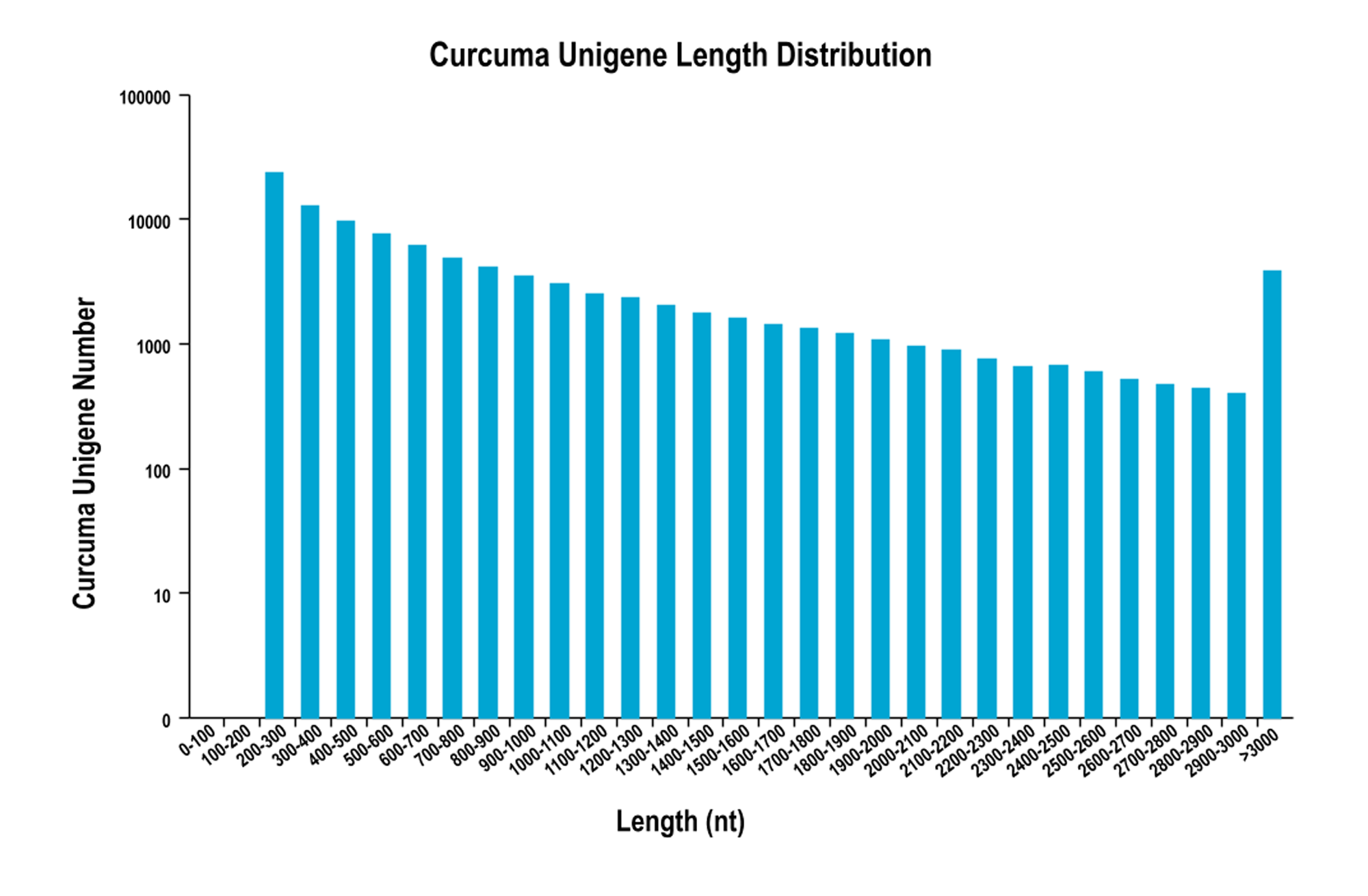

Supplement: S1 Fig — (TIF) [file pone.0242776.s001.tif]

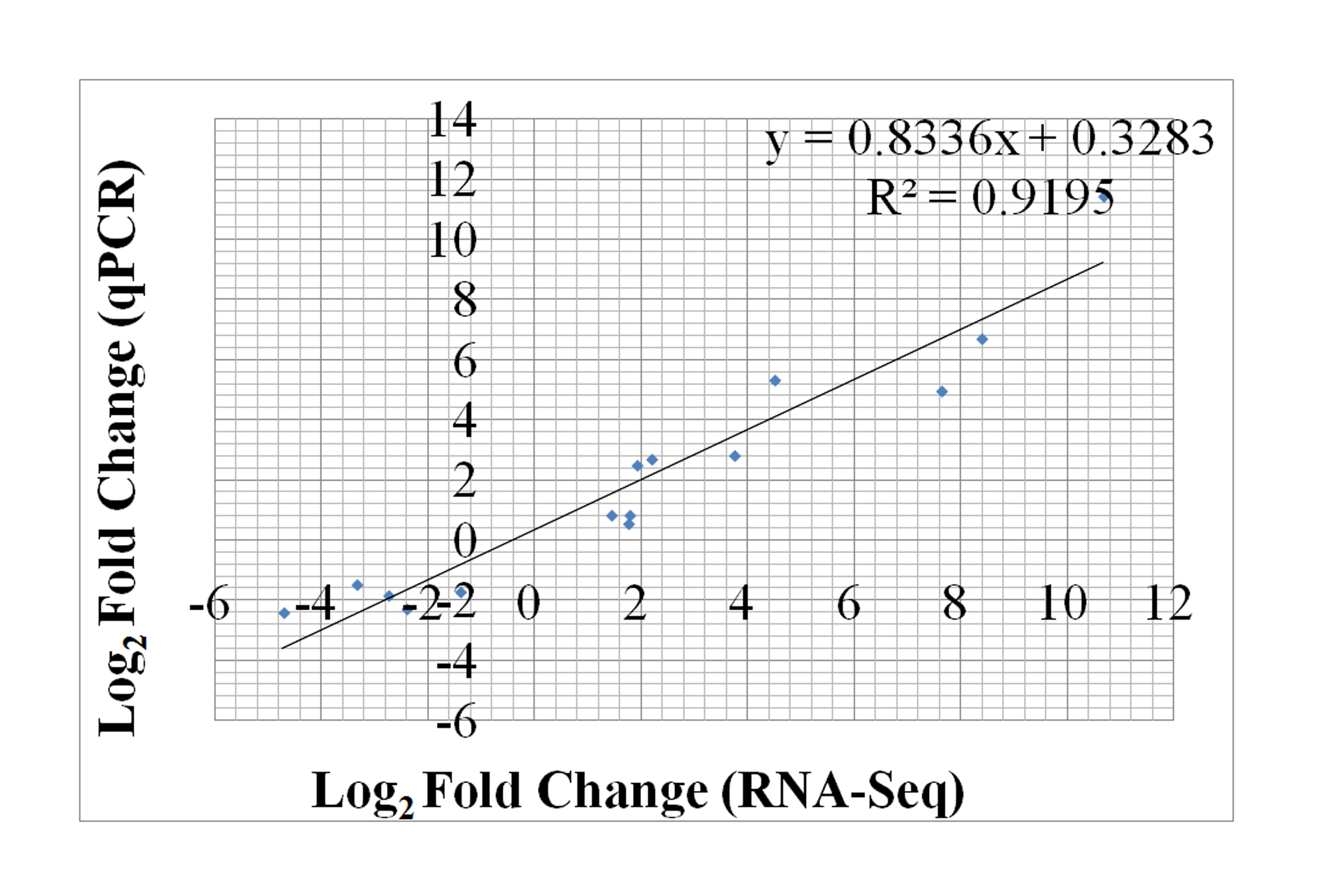

Supplement: S2 Fig — log2Fold Change [Wenzhou (WZ)/Haikou (HK)]. (TIF) [file pone.0242776.s002.tif]

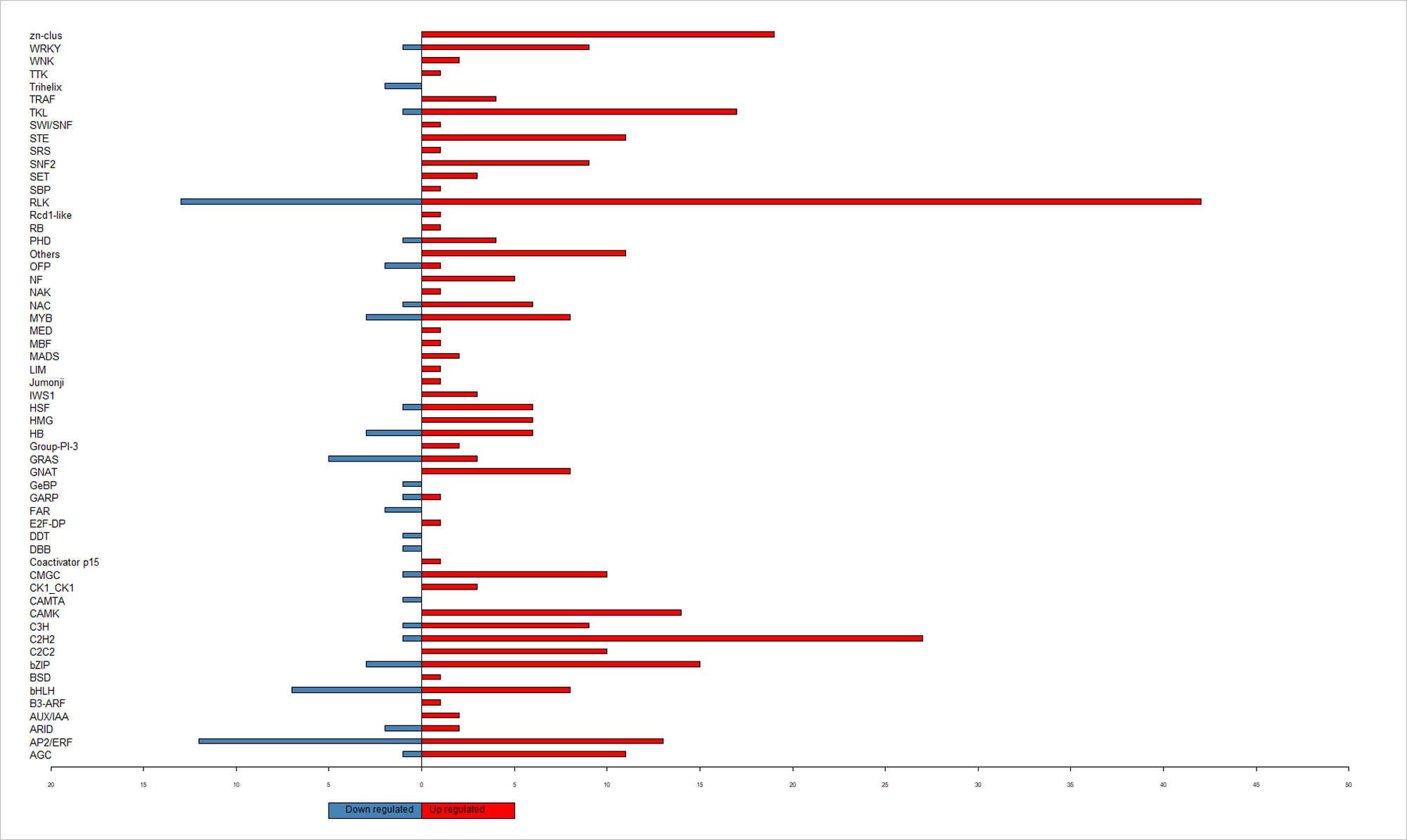

Supplement: S3 Fig — (TIF) [file pone.0242776.s003.tif]

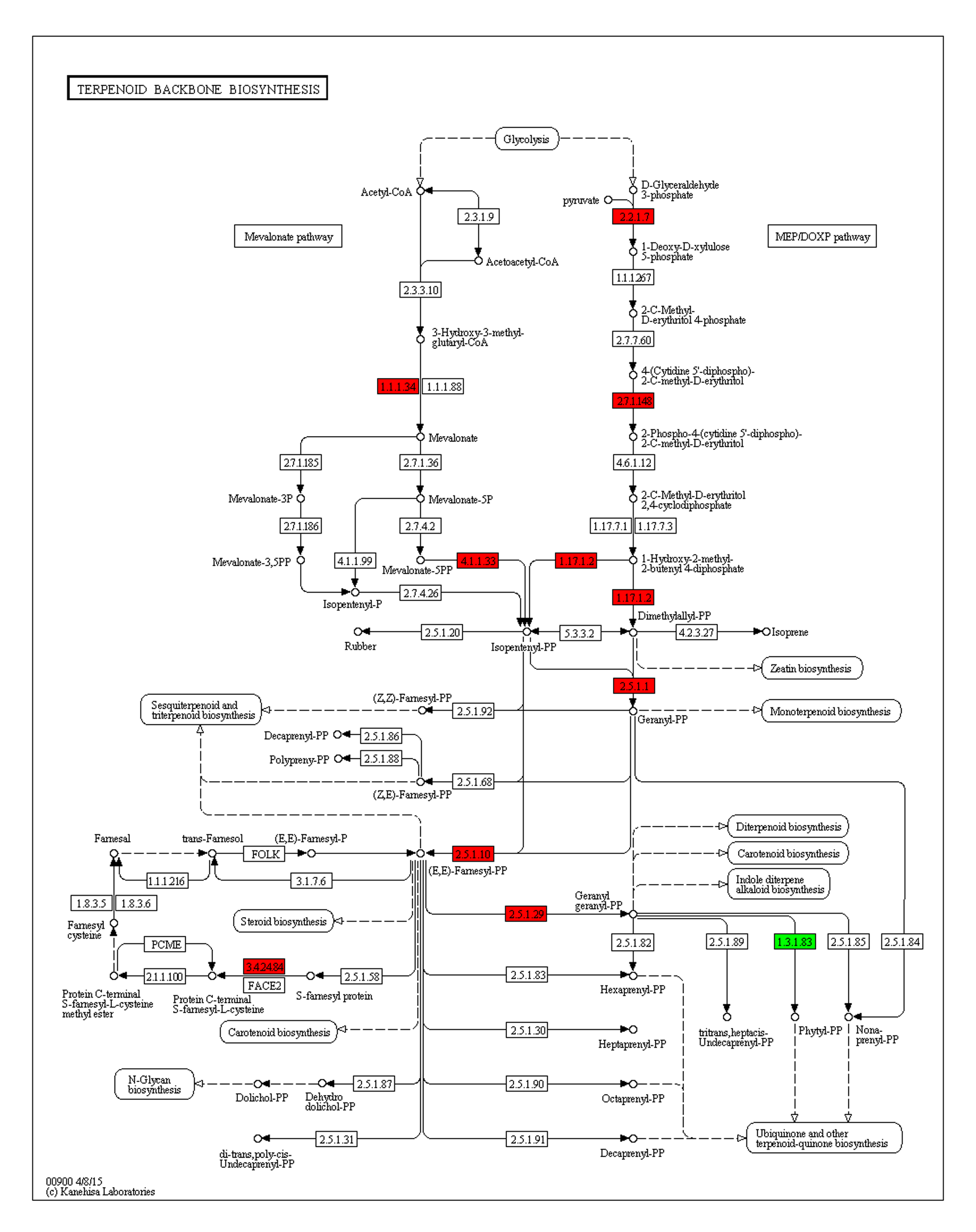

Supplement: S4 Fig — (TIF) [file pone.0242776.s004.tif]

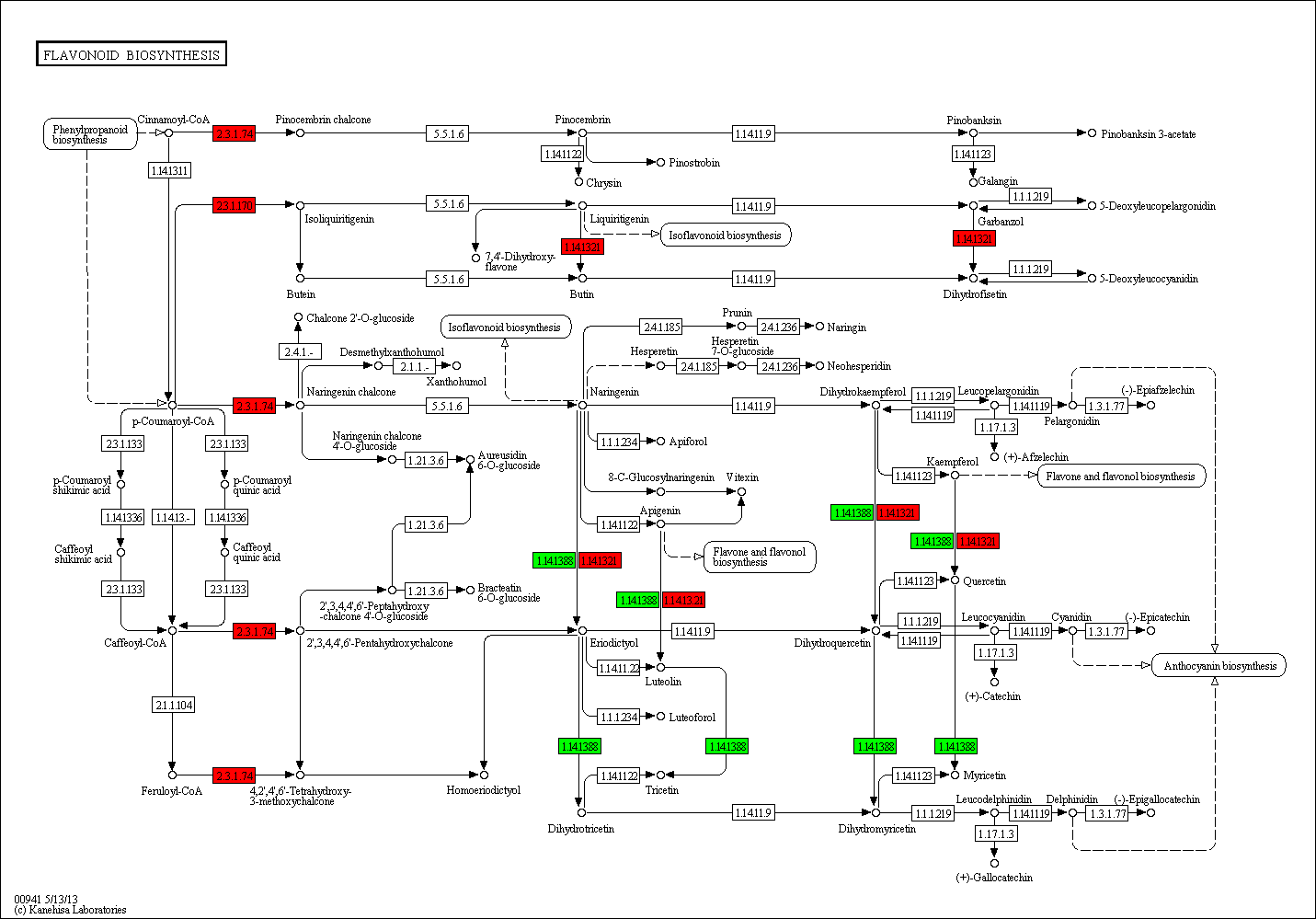

Supplement: S5 Fig — (TIF) [file pone.0242776.s005.tif]

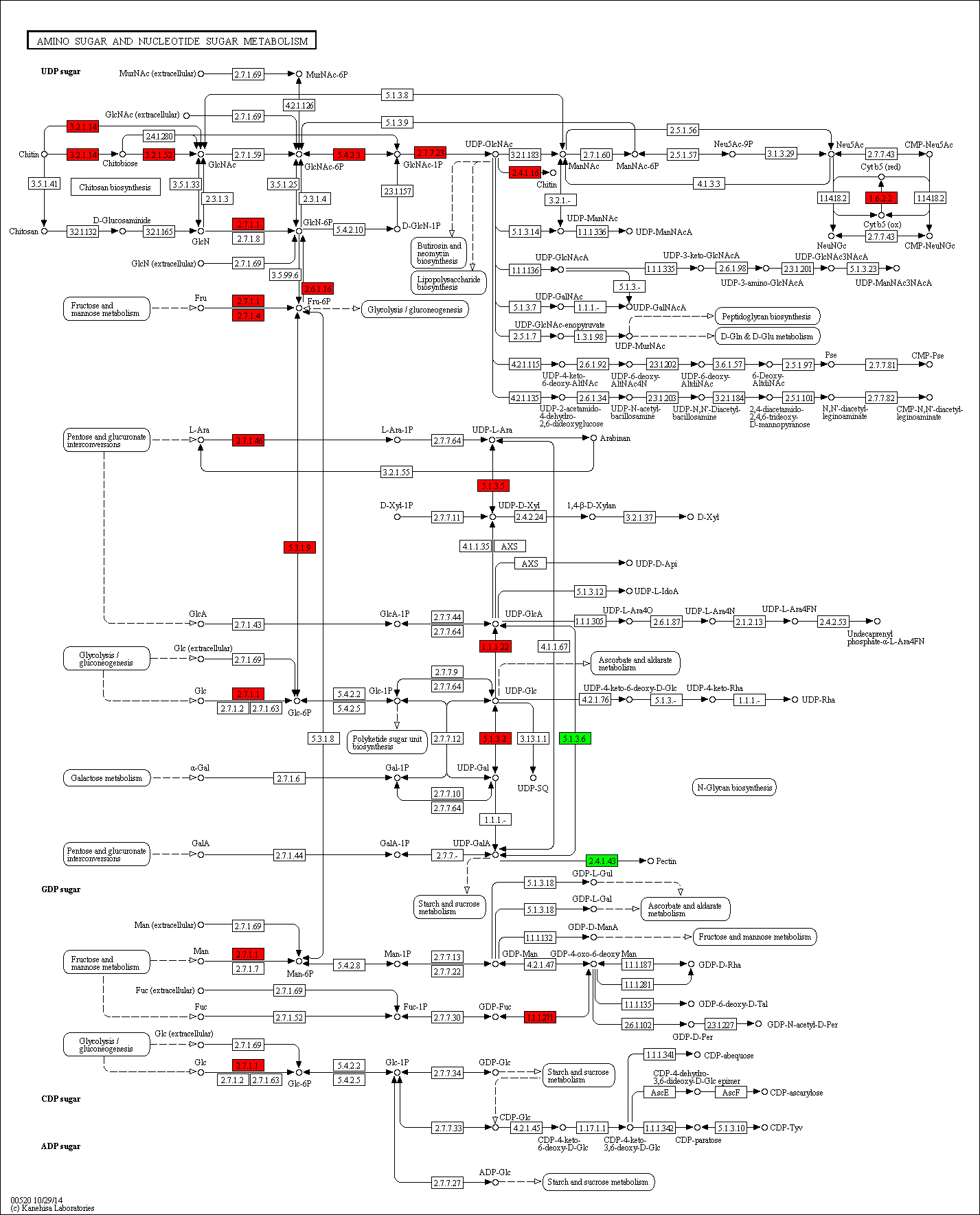

Supplement: S6 Fig — (TIF) [file pone.0242776.s006.tif]

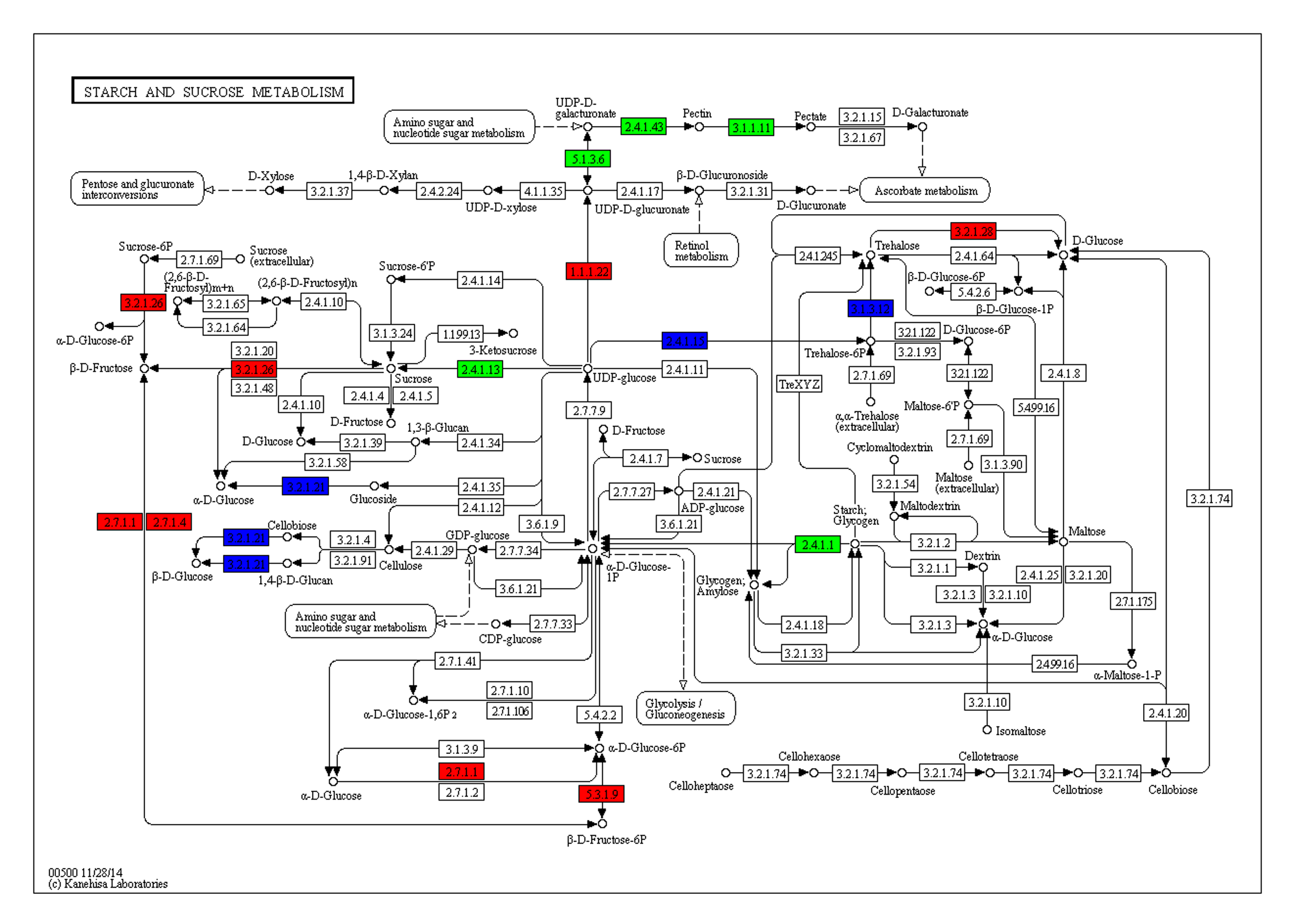

Supplement: S7 Fig — (TIF) [file pone.0242776.s007.tif]

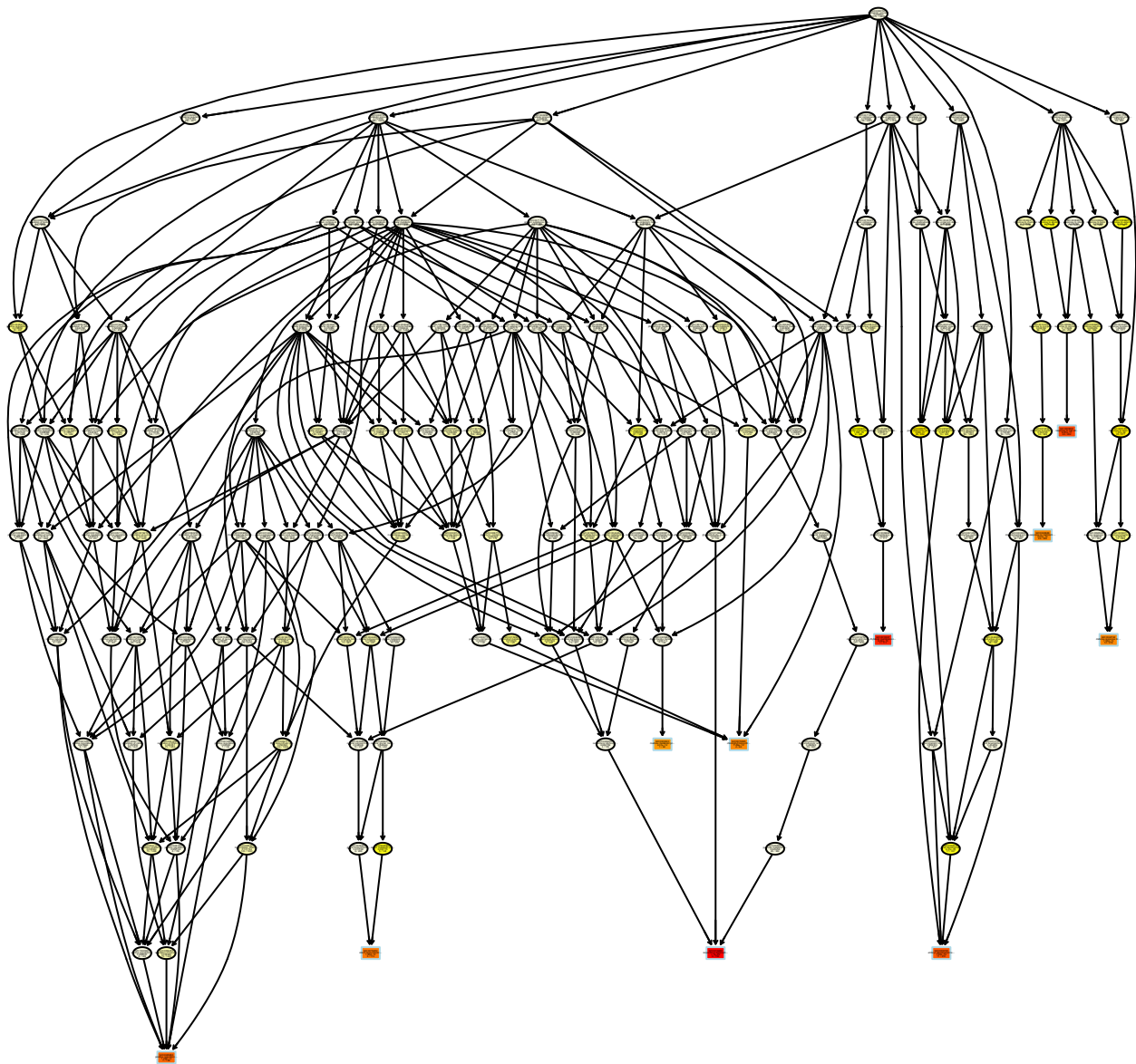

Supplement: S1 File — The node size is proportional to the number of targets in the GO category. Node color represents enriched significance; a deeper color represents a higher significance [a1, a2, and a3: total (topGO_BP, topGO_CC, and topGO_MF); b1, b2, and b3: down-regulated (topGO_BP, topGO_CC, and topGO_MF); c1, c2, and c3: up-regulated (topGO_BP, topGO_CC, and topGO_MF)]. (ZIP) [file pone.0242776.s019.zip › S1_File/S1a1_File.pdf]

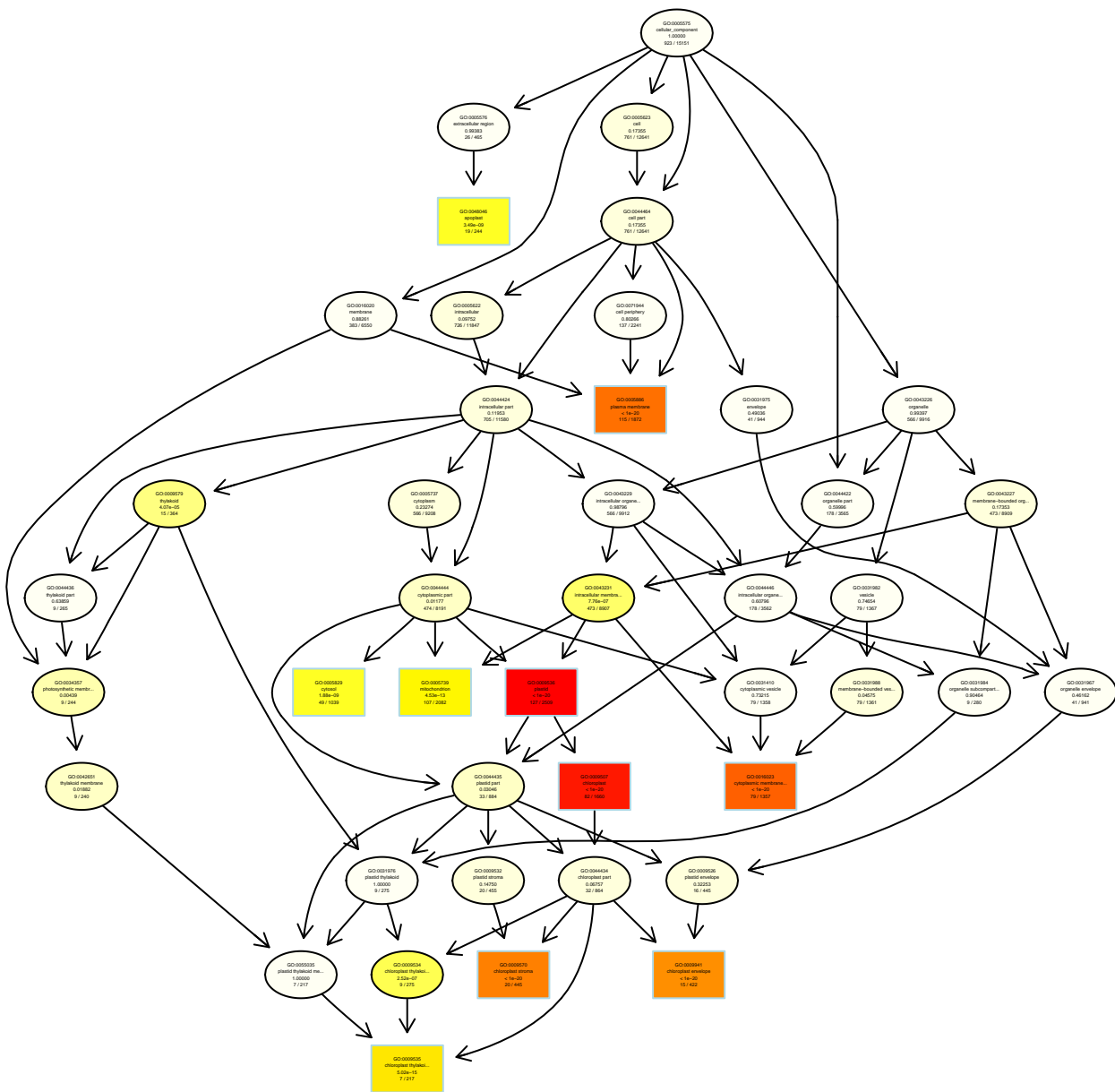

Supplement: S1 File — The node size is proportional to the number of targets in the GO category. Node color represents enriched significance; a deeper color represents a higher significance [a1, a2, and a3: total (topGO_BP, topGO_CC, and topGO_MF); b1, b2, and b3: down-regulated (topGO_BP, topGO_CC, and topGO_MF); c1, c2, and c3: up-regulated (topGO_BP, topGO_CC, and topGO_MF)]. (ZIP) [file pone.0242776.s019.zip › S1_File/S1a2_File.pdf]

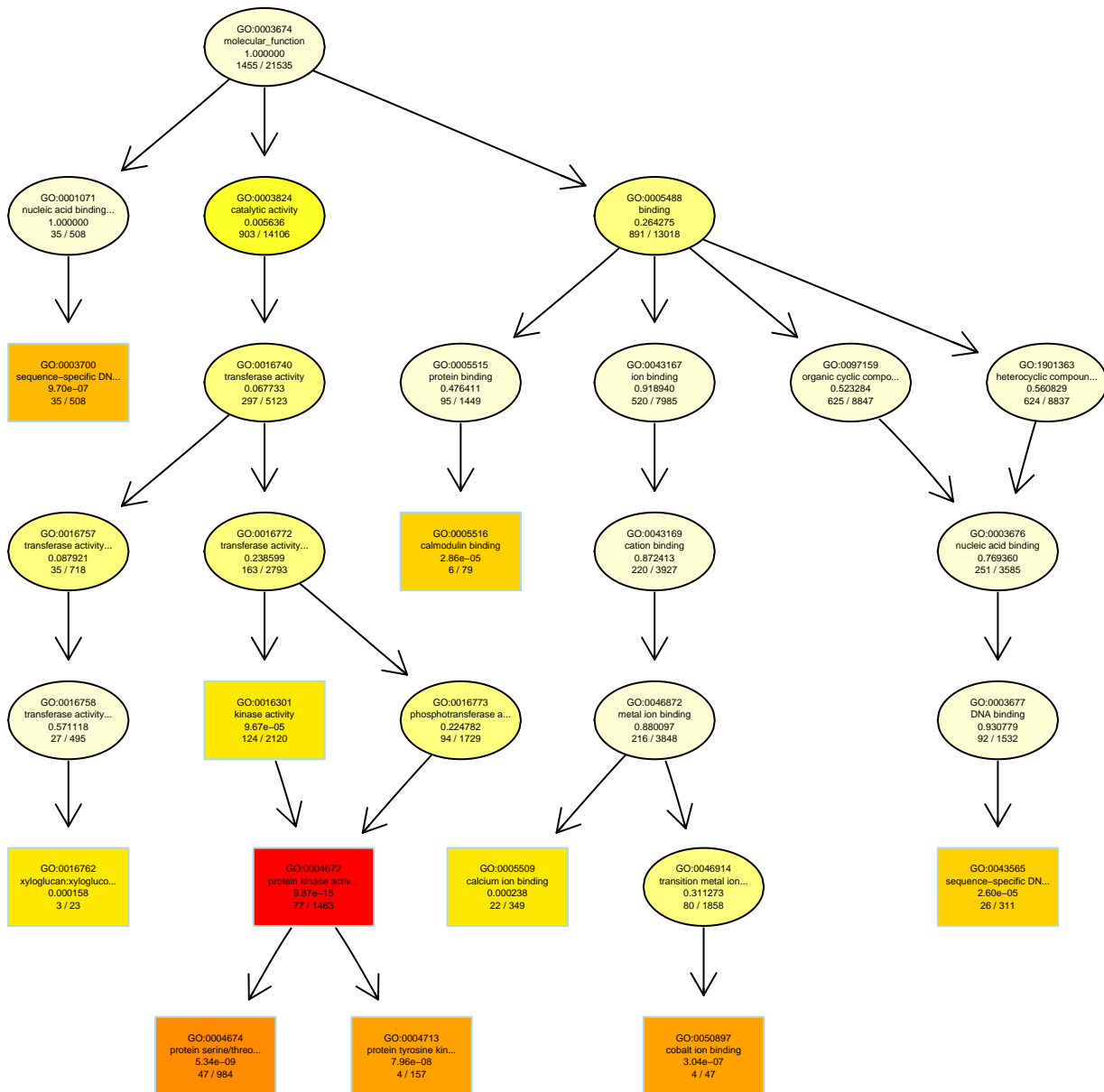

Supplement: S1 File — The node size is proportional to the number of targets in the GO category. Node color represents enriched significance; a deeper color represents a higher significance [a1, a2, and a3: total (topGO_BP, topGO_CC, and topGO_MF); b1, b2, and b3: down-regulated (topGO_BP, topGO_CC, and topGO_MF); c1, c2, and c3: up-regulated (topGO_BP, topGO_CC, and topGO_MF)]. (ZIP) [file pone.0242776.s019.zip › S1_File/S1a3_File.pdf]

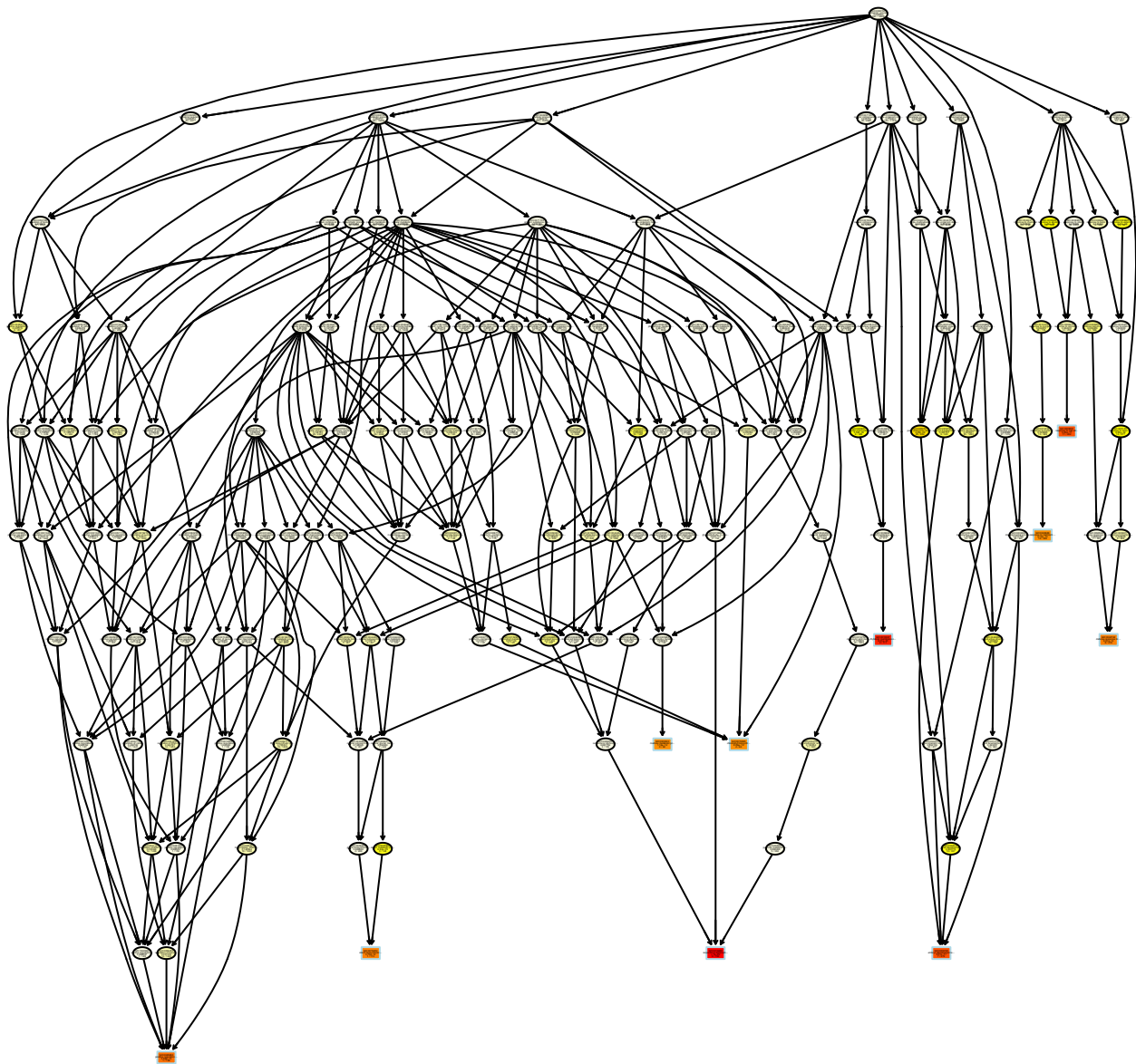

Supplement: S1 File — The node size is proportional to the number of targets in the GO category. Node color represents enriched significance; a deeper color represents a higher significance [a1, a2, and a3: total (topGO_BP, topGO_CC, and topGO_MF); b1, b2, and b3: down-regulated (topGO_BP, topGO_CC, and topGO_MF); c1, c2, and c3: up-regulated (topGO_BP, topGO_CC, and topGO_MF)]. (ZIP) [file pone.0242776.s019.zip › S1_File/S1b1_File.pdf]

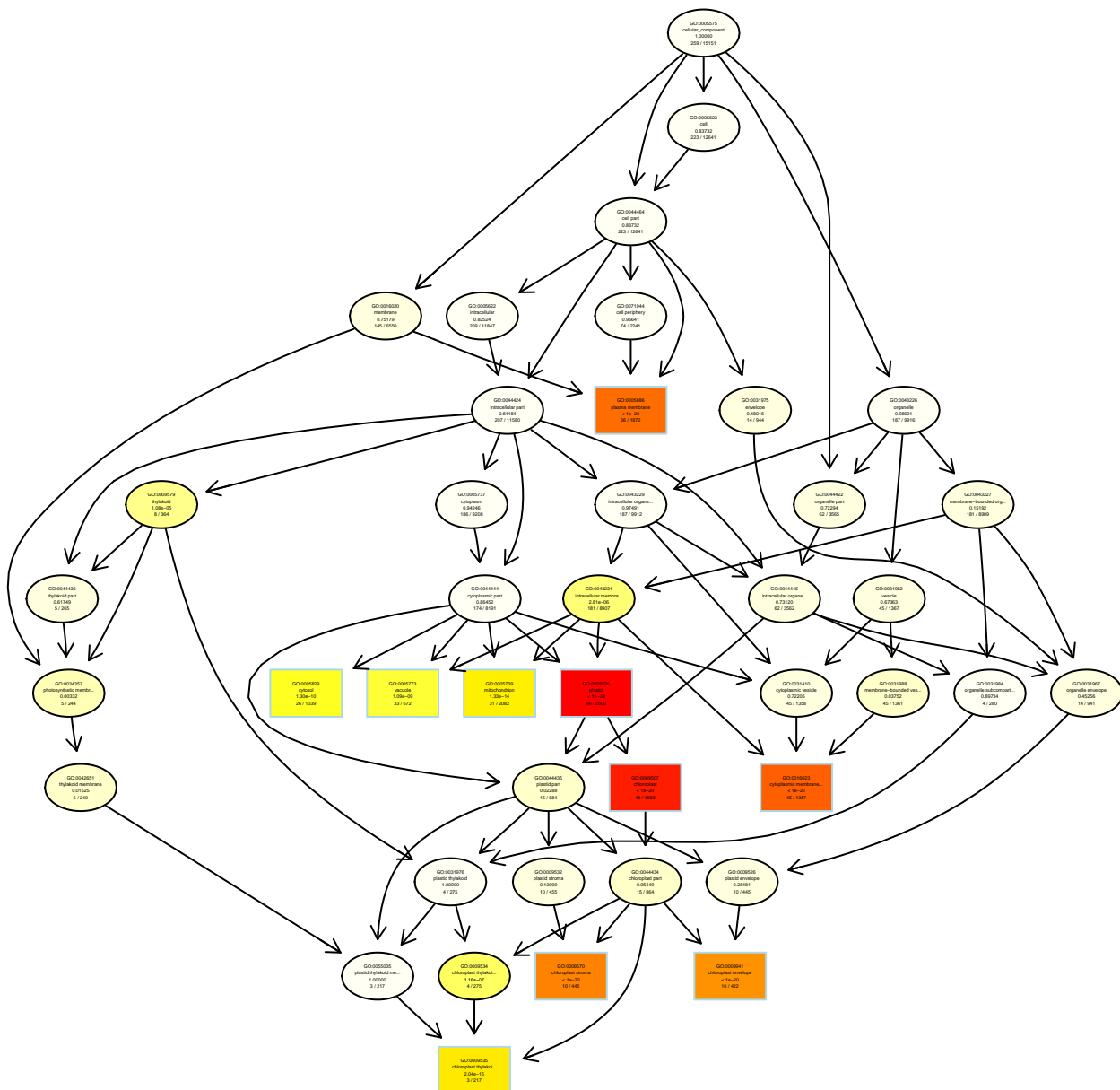

Supplement: S1 File — The node size is proportional to the number of targets in the GO category. Node color represents enriched significance; a deeper color represents a higher significance [a1, a2, and a3: total (topGO_BP, topGO_CC, and topGO_MF); b1, b2, and b3: down-regulated (topGO_BP, topGO_CC, and topGO_MF); c1, c2, and c3: up-regulated (topGO_BP, topGO_CC, and topGO_MF)]. (ZIP) [file pone.0242776.s019.zip › S1_File/S1b2_File.pdf]

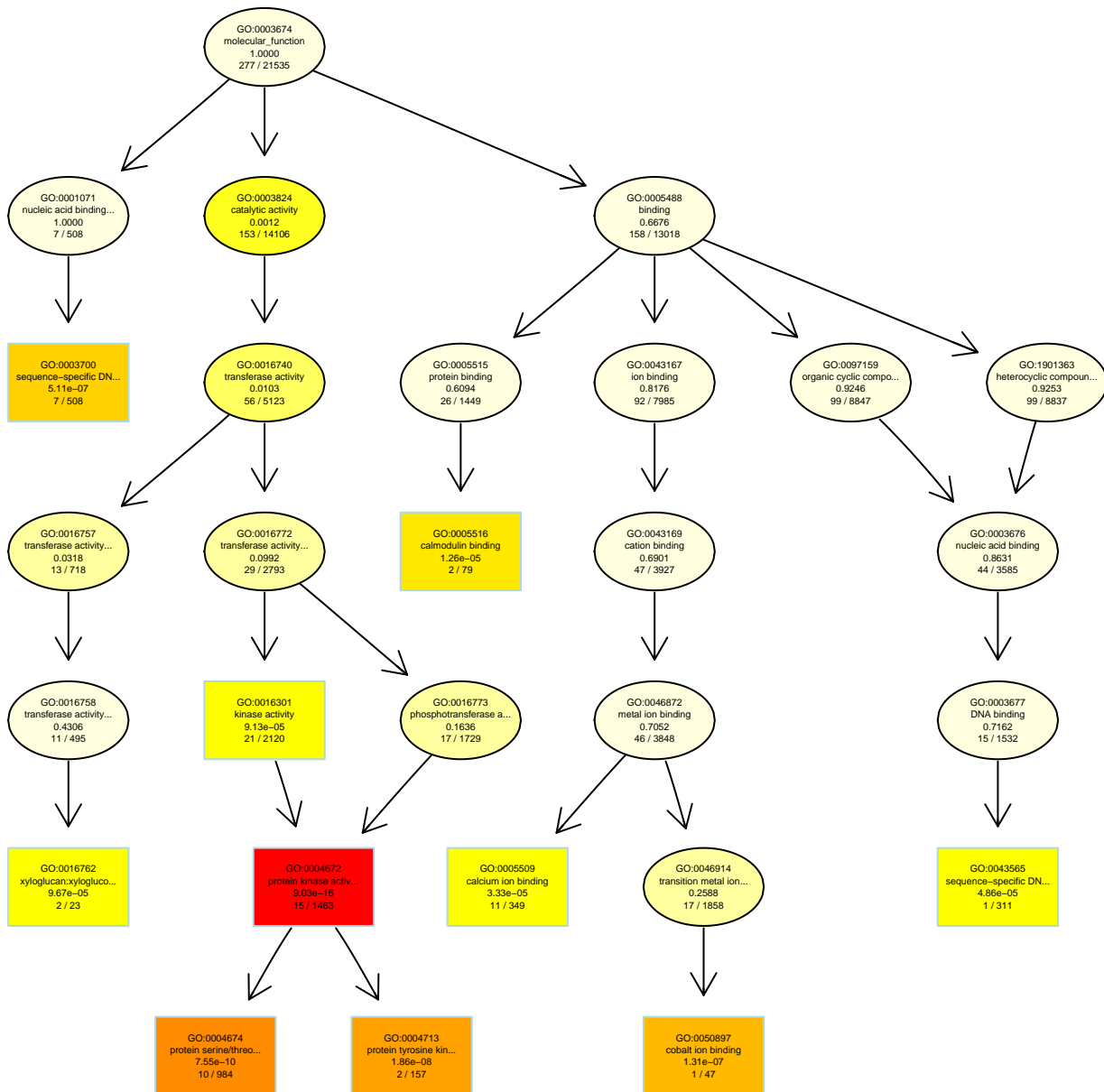

Supplement: S1 File — The node size is proportional to the number of targets in the GO category. Node color represents enriched significance; a deeper color represents a higher significance [a1, a2, and a3: total (topGO_BP, topGO_CC, and topGO_MF); b1, b2, and b3: down-regulated (topGO_BP, topGO_CC, and topGO_MF); c1, c2, and c3: up-regulated (topGO_BP, topGO_CC, and topGO_MF)]. (ZIP) [file pone.0242776.s019.zip › S1_File/S1b3_File.pdf]

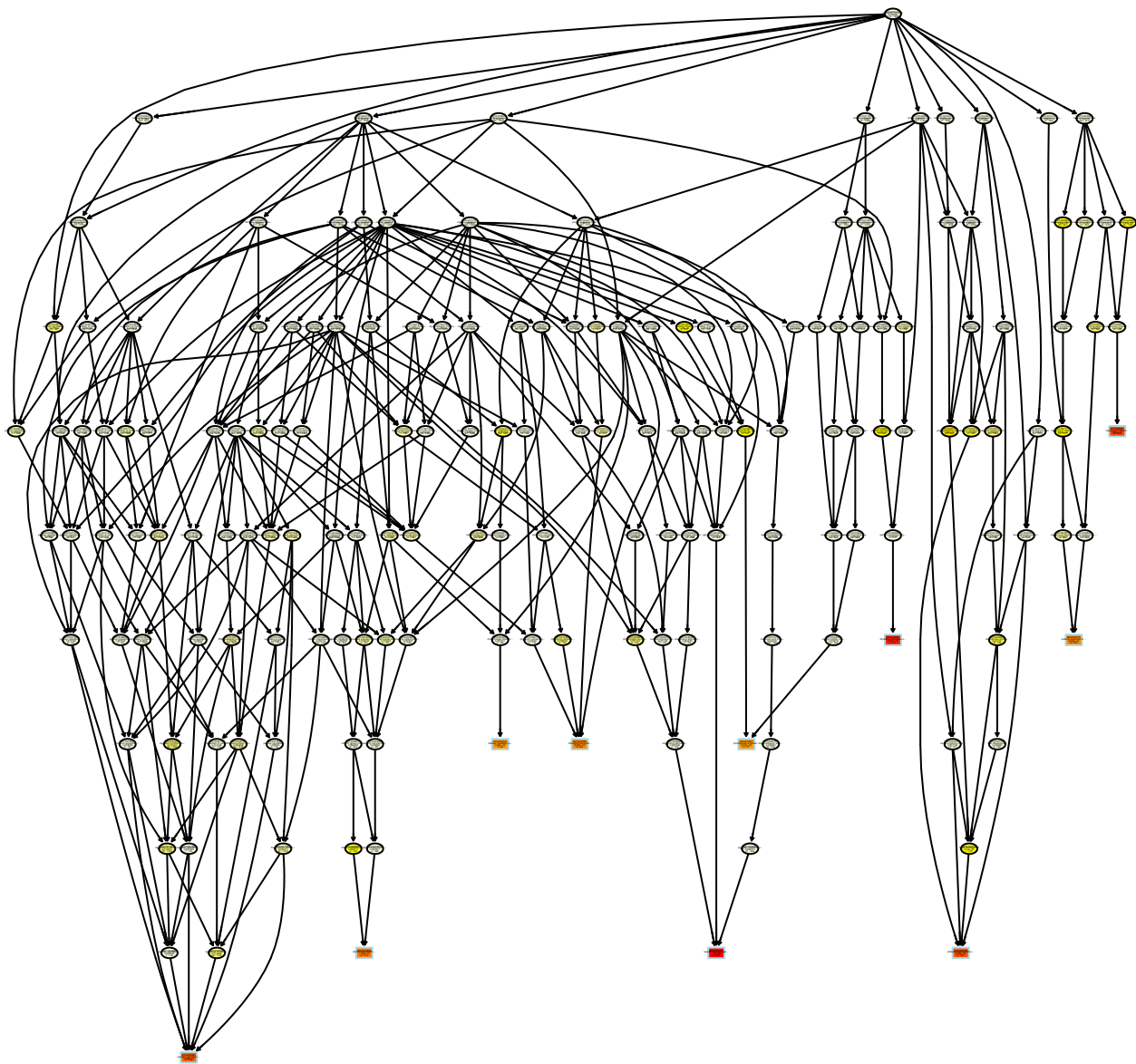

Supplement: S1 File — The node size is proportional to the number of targets in the GO category. Node color represents enriched significance; a deeper color represents a higher significance [a1, a2, and a3: total (topGO_BP, topGO_CC, and topGO_MF); b1, b2, and b3: down-regulated (topGO_BP, topGO_CC, and topGO_MF); c1, c2, and c3: up-regulated (topGO_BP, topGO_CC, and topGO_MF)]. (ZIP) [file pone.0242776.s019.zip › S1_File/S1c1_File.pdf]

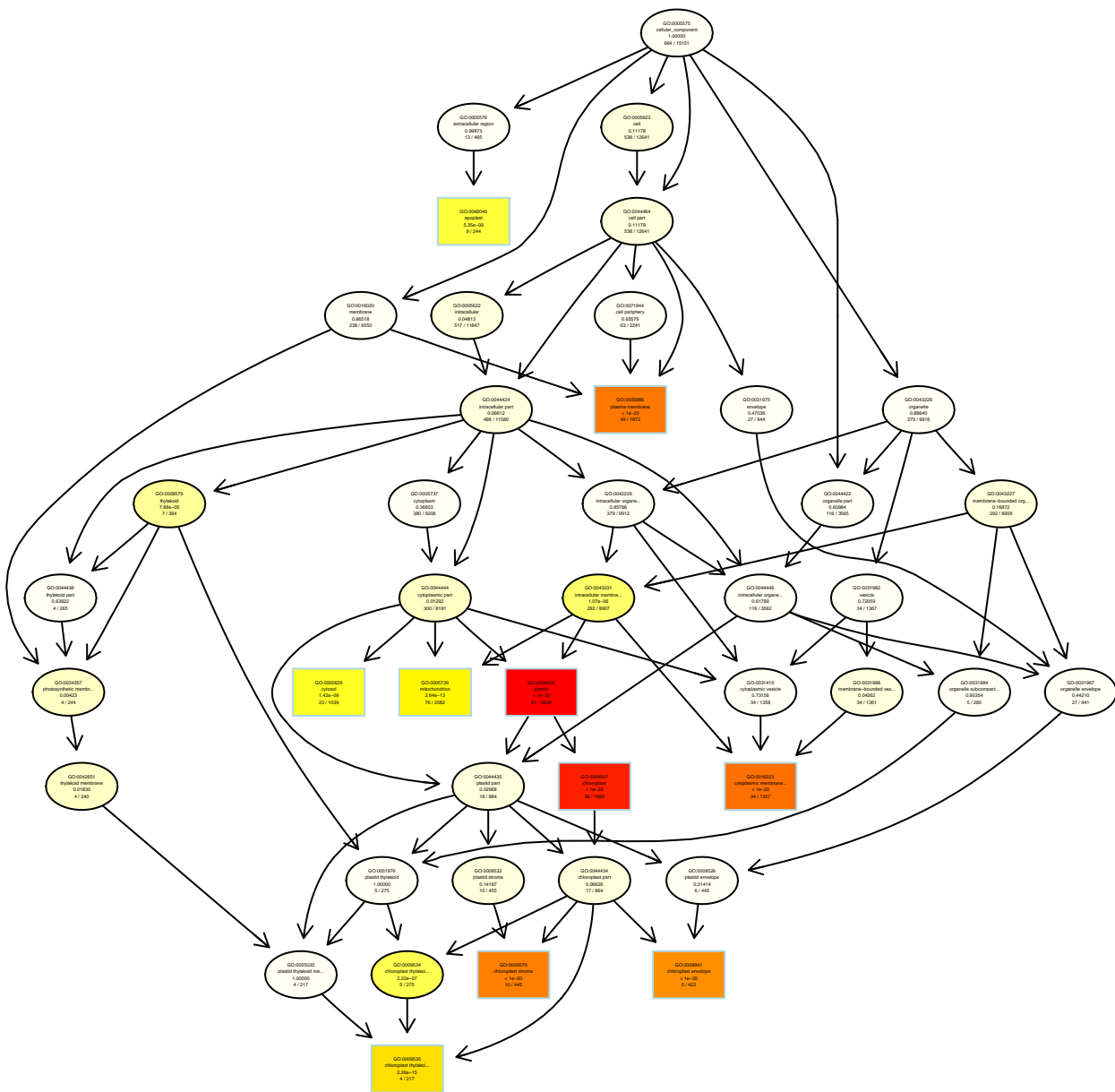

Supplement: S1 File — The node size is proportional to the number of targets in the GO category. Node color represents enriched significance; a deeper color represents a higher significance [a1, a2, and a3: total (topGO_BP, topGO_CC, and topGO_MF); b1, b2, and b3: down-regulated (topGO_BP, topGO_CC, and topGO_MF); c1, c2, and c3: up-regulated (topGO_BP, topGO_CC, and topGO_MF)]. (ZIP) [file pone.0242776.s019.zip › S1_File/S1c2_File.pdf]

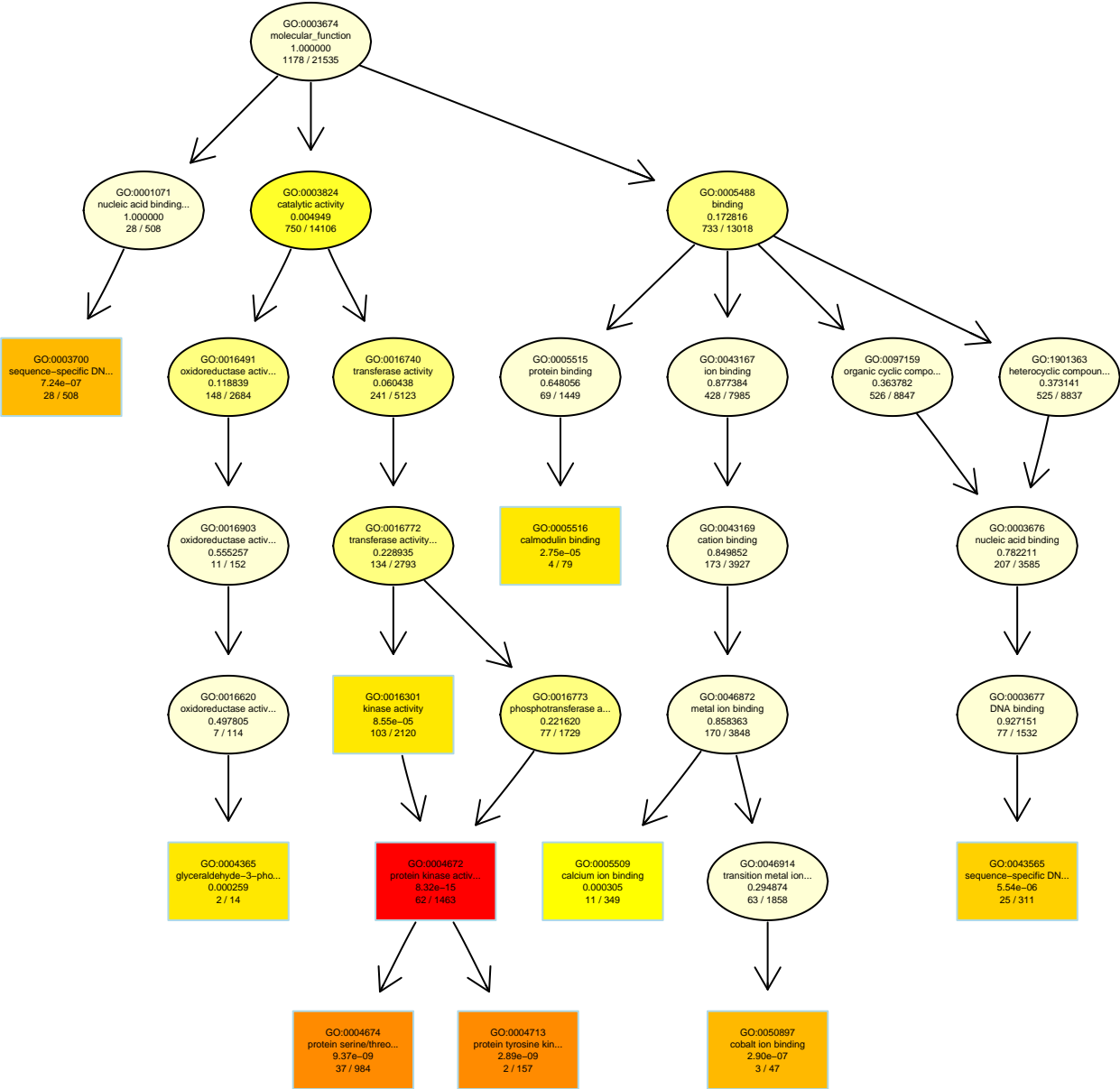

Supplement: S1 File — The node size is proportional to the number of targets in the GO category. Node color represents enriched significance; a deeper color represents a higher significance [a1, a2, and a3: total (topGO_BP, topGO_CC, and topGO_MF); b1, b2, and b3: down-regulated (topGO_BP, topGO_CC, and topGO_MF); c1, c2, and c3: up-regulated (topGO_BP, topGO_CC, and topGO_MF)]. (ZIP) [file pone.0242776.s019.zip › S1_File/S1c3_File.pdf]
