# Supplementary material for: Transcriptome analysis of Curcuma wenyujin from Haikou and Wenzhou, and a comparison of the main constituents and related genes of Rhizoma Curcumae
Source: PLoS One. 2020 Nov 30;15(11):e0242776. doi: 10.1371/journal.pone.0242776 (PMC7703983; doi:10.1371/journal.pone.0242776)
Supplement: Attachment — Submitted filename: PONE-D-19-24237_reviewer.pdf [file pone.0242776.s020.pdf]

PLOS ONE

Transcriptome analysis of Curcumawenyujin from Haikou and Wenzhou and a comparison of the main substances and related genes of Rhizoma Curcumae

--Manuscript Draft--

**Manuscript Number:**	PONE-D-19-24237
**Article Type:**	Research Article
**Full Title:**	Transcriptome analysis of Curcumawenyujin from Haikou and Wenzhou and a comparison of the main substances and related genes of Rhizoma Curcumae
**Short Title:**	a comparison of the main substances and related genes of Rhizoma Curcumae
**Corresponding Author:**	Lu Lilan<br>Chinese Academy of Medical Sciences & Peking Union Medical College<br>Haikou, CHINA
**Keywords:**	Rhizoma Curcumae; transcriptome; main substances; related genes; two producing areas
**Abstract:**	For over a thousand years, Rhizoma Curcumae (known as Ezhu), a Chinese herbal medicine, has been used to eradicate blood stasis and relieve ache. Curcuma wenyujin is considered the best source of Rhizoma Curcumae and is mainly grown in Wenzhou, China. In this study, the transcript profiles of Rhizoma Curcumae Wenyujin in the traditional (Wenzhou) and introduced (Haikou) production areas were investigated using Illumina and RNA-sequencing (RNA-seq). Changes in the main components of volatile oil terpene, curcumin, polysaccharide, and starch substances of Rhizoma Curcumae, and the related genes in the corresponding pathways of C. wenyujin from these two producing areas, were compared and investigated. The essential oil (2.05 %), curcumin (1.46 %), and polysaccharides (8.90 %) in rhizomes from Wenzhou were higher than those in rhizomes from Haikou (1.60 %, 0.91 %, and 6.15 %, respectively); the starch content of rhizomes from Wenzhou (17.0 %) was lower than that of rhizomes from Haikou (23.8 %). Oil components significantly varied between Haikou and Wenzhou rhizomes. Curzerene (32.34 %), curdione (21.35 %), and germacrene B (9.39 %) were the primary components of the essential oil from C. wenyujin rhizomes in Wenzhou . The major essential oil components in rhizomes from Haikou were curzerene (20.13 %), curdione (14.73 %), and cineole (9.76 %). Transcriptome and qRT-PCR (quantitative real-time PCR) analyses showed that expression of genes between Wenzhou and Haikou rhizomes varied considerably. Compared to Haikou, the expression of volatile oil terpene, curcumin, and polysaccharide pathway-related genes in Wenzhou was significantly up-regulated and the expression of starch-associated genes was significantly down-regulated. We found that the difference in contents of the essential oil terpene, curcumin, polysaccharides, and starch of the rhizomes from the two areas could be explained by the differences in expression of the related genes.
**Order of Authors:**	Lu Lilan
	Liu Peiwei
	Zhang Yuxiu
	Wang Caixia
	Feng Jian
	Wei Jianhe
**Additional Information:**	
**Question**	**Response**
**Financial Disclosure**<br><br>Enter a financial disclosure statement that describes the sources of funding for the	YES，Hainan province Key Scientific and Technological Research and Development Special Project (Grant No: ZDKJ2016006)，The funders had a role in study design, data collection and analysis, decision to publish, or preparation of the manuscript.

Powered by Editorial Manager® and ProduXion Manager® from Aries Systems Corporation

work included in this submission. Review the submission guidelines for detailed requirements. View published research articles from *PLOS ONE* for specific examples.<br><br>This statement is required for submission and **will appear in the published article** if the submission is accepted. Please make sure it is accurate.<br><br>**Unfunded studies**<br>Enter: *The author(s) received no specific funding for this work.*<br><br>**Funded studies**<br>Enter a statement with the following details:<br>• Initials of the authors who received each award<br>• Grant numbers awarded to each author<br>• The full name of each funder<br>• URL of each funder website<br>• Did the sponsors or funders play any role in the study design, data collection and analysis, decision to publish, or preparation of the manuscript?<br>• **NO** - Include this sentence at the end of your statement: *The funders had no role in study design, data collection and analysis, decision to publish, or preparation of the manuscript.*<br>• **YES** - Specify the role(s) played.<br><br>* typeset	
**Competing Interests**<br><br>Use the instructions below to enter a competing interest statement for this submission. On behalf of all authors, disclose any competing interests that could be perceived to bias this work—acknowledging all financial support and any other relevant financial or non-financial competing interests.<br><br>This statement **will appear in the published article** if the submission is accepted. Please make sure it is accurate. View published research articles from *PLOS ONE* for specific examples.	The authors have declared that no competing interests exist.

**NO authors have competing interests**<br><br>Enter: *The authors have declared that no competing interests exist.*<br><br>**Authors with competing interests**<br><br>Enter competing interest details beginning with this statement:<br><br>*I have read the journal's policy and the authors of this manuscript have the following competing interests: [insert competing interests here]*<br><br>* typeset	
**Ethics Statement**<br><br>Enter an ethics statement for this submission. This statement is required if the study involved:<br><br>• Human participants<br>• Human specimens or tissue<br>• Vertebrate animals or cephalopods<br>• Vertebrate embryos or tissues<br>• Field research<br><br>Write "N/A" if the submission does not require an ethics statement.<br><br>General guidance is provided below. Consult the submission guidelines for detailed instructions. **Make sure that all information entered here is included in the Methods section of the manuscript.**	N/A

**Format for specific study types**<br><br>**Human Subject Research (involving human participants and/or tissue)**<br>• Give the name of the institutional review board or ethics committee that approved the study<br>• Include the approval number and/or a statement indicating approval of this research<br>• Indicate the form of consent obtained (written/oral) or the reason that consent was not obtained (e.g. the data were analyzed anonymously)<br><br>**Animal Research (involving vertebrate animals, embryos or tissues)**<br>• Provide the name of the Institutional Animal Care and Use Committee (IACUC) or other relevant ethics board that reviewed the study protocol, and indicate whether they approved this research or granted a formal waiver of ethical approval<br>• Include an approval number if one was obtained<br>• If the study involved *non-human primates*, add *additional details* about animal welfare and steps taken to ameliorate suffering<br>• If anesthesia, euthanasia, or any kind of animal sacrifice is part of the study, include briefly which substances and/or methods were applied<br><br>**Field Research**<br><br>Include the following details if this study involves the collection of plant, animal, or other materials from a natural setting:<br>• Field permit number<br>• Name of the institution or relevant body that granted permission	
**Data Availability**<br><br>Authors are required to make all data underlying the findings described fully available, without restriction, and from the time of publication. PLOS allows rare exceptions to address legal and ethical concerns. See the PLOS Data Policy and FAQ for detailed information.	Yes - all data are fully available without restriction

Powered by Editorial Manager® and ProduXion Manager® from Aries Systems Corporation

A Data Availability Statement describing where the data can be found is required at submission. Your answers to this question constitute the Data Availability Statement and **will be published in the article**, if accepted.<br><br>**Important:** Stating 'data available on request from the author' is not sufficient. If your data are only available upon request, select 'No' for the first question and explain your exceptional situation in the text box.<br><br>Do the authors confirm that all data underlying the findings described in their manuscript are fully available without restriction?	
**Describe where the data may be found in full sentences. If you are copying our sample text, replace any instances of XXX with the appropriate details.**<br><br>• If the data are **held or will be held in a public repository**, include URLs, accession numbers or DOIs. If this information will only be available after acceptance, indicate this by ticking the box below. For example: *All XXX files are available from the XXX database (accession number(s) XXX, XXX.)*.<br>• If the data are all contained **within the manuscript and/or Supporting Information files**, enter the following: *All relevant data are within the manuscript and its Supporting Information files.*<br>• If neither of these applies but you are able to provide **details of access elsewhere**, with or without limitations, please do so. For example:<br><br>*Data cannot be shared publicly because of [XXX]. Data are available from the XXX Institutional Data Access / Ethics Committee (contact via XXX) for researchers who meet the criteria for access to confidential data.*<br><br>*The data underlying the results presented in the study are available from (include the name of the third party*	All relevant data are within the manuscript and its Supporting Information files

and contact information or URL).<br>• This text is appropriate if the data are owned by a third party and authors do not have permission to share the data.<br><br>* typeset	
Additional data availability information:	Tick here if your circumstances are not covered by the questions above and you need the journal's help to make your data available.

Powered by Editorial Manager® and ProduXion Manager® from Aries Systems Corporation

Manuscript
Click here to download Manuscript manuscript-3-1-re1.docx

Transcriptome analysis of *Curcuma wenyujin* from Haikou and Wenzhou and a comparison of the main substances and related genes of *Rhizoma Curcumae*

Lilan Lu[1], Peiwei Liu [1], Yuxiu Zhang [1], Caixia Wang [2], Jian Feng [1], Jianhe Wei [3*]

[1] Haikou Provincial Key Laboratory of Resources Conservation and Development of Southern Medicine, Haikou Branch of the Institute of Medicinal Plant Development, Chinese Academy of Medical Sciences and Peking Union Medical College, Haikou, China

[2] Institute of Agricultural Environment and Soil, Hainan Academy of Agricultural Sciences, Haikou, China

[3] Institute of Medicinal Plant Development, Chinese Academy of Medical Sciences & Peking Union Medical College, Beijing, China

* Corresponding author

E-mail: wjianh@263.net (JWH)

Abstract

For over a thousand years, Rhizoma Curcumae (known as Ezhu), a Chinese herbal medicine, has been used to eradicate blood stasis and relieve ache. *Curcuma wenyujin* is considered the best source of Rhizoma Curcumae and is mainly grown in Wenzhou, China. In this study, the transcript profiles of Rhizoma Curcumae Wenyujin in the traditional (Wenzhou) and introduced (Haikou) production areas were investigated using Illumina and RNA-sequencing (RNA-seq). Changes in the main components of volatile oil terpene, curcumin, polysaccharide, and starch substances of Rhizoma Curcumae, and the related genes in the corresponding pathways of *C. wenyujin* from these two producing areas, were compared and investigated. The essential oil (2.05 %), curcumin (1.46 %), and polysaccharides (8.90 %) in rhizomes from Wenzhou were higher than those in rhizomes from Haikou (1.60 %, 0.91 %, and 6.15 %, respectively); the starch content of rhizomes from Wenzhou (17.0 %) was lower than that of rhizomes from Haikou (23.8 %). Oil components significantly varied between Haikou and Wenzhou rhizomes. Curzerene (32.34 %), curdione (21.35 %), and germacrene B (9.39 %) were the primary components of the essential oil from *C. wenyujin* rhizomes in Wenzhou. The major essential oil components in rhizomes from Haikou were curzerene (20.13 %), curdione (14.73 %), and cineole (9.76 %). Transcriptome and ~~qRT-PCR (quantitative real-time PCR)~~ analyses showed that expression of genes between Wenzhou and Haikou rhizomes varied considerably. Compared to Haikou, the expression of

volatile oil terpene, curcumin, and polysaccharide pathway-related genes in Wenzhou was significantly up-regulated and the expression of starch-associated genes was significantly down-regulated. We found that the difference in contents of the essential oil terpene, curcumin, polysaccharides, and starch of the rhizomes from the two areas could be explained by the differences in expression of the related genes.

Introduction

Curcuma ~~(*Curcuma zedoaria* (*Christm.*) *Roscoe.*)~~ is a genus that has more than 70 rhizome-producing species worldwide. There are approximately 20 kinds of *Curcuma* species in China, a small number of which are used in Chinese herbal medicines and food products. Rhizoma Curcuma is the dried rhizome of *Curcuma kwangsiensis* S. G. Lee & C. F. Liang, *Curcuma phaeocaulis* Valeton, and *Curcuma wenyujin* Y. H. Chen & C. Ling [1]. In practice, however, other non-rhizomatous sections from these three *Curcuma* species, and other closely related plants from the *Curcuma* genus, are also applied to herbal medicines and food products, but for different therapeutic uses. *Curcuma* essential oils are regarded as active ingredients and have antiviral and antitumor properties [2-5]. The main constituents of the essential oils in *Curcumae* rhizomes are curcumol, germacrone, and curdione, which account for most of the known pharmacological effects. These constituents are usually used as quality control markers for the essential oils [6-8]. Among the different sources of Rhizoma Curcumae, *C. wenyujin* from Wenzhou, in the Zhejiang province in China, is considered to be the best source, because it has distinctive regional

characteristics and a high medicinal value; it is mainly produced in Ruian, Wenzhou. Rhizoma Curcumae, also known as “*e zhu*” in Chinese, is an herbal medicine, food component, and cosmetic ingredient, and has been used for more than a thousand years [9-11]. The main active ingredients of *C. wenyujin* are volatile oil and curcumin [12,13] The volatile oil is composed of terpenoids and sesquiterpene derivatives, which have been reported to have anti-inflammatory, anti-tumor, lipid-regulating, anti-oxidation, antiviral, antibacterial, and other therapeutic effects [14-17].

C. wenyujin rhizomes are mainly cultivated in China; however, the quality of rhizomes changes significantly according to ~~species~~ variety, cultivation region, cultivation techniques, and extraction methods [11,18,19]. *C. wenyujin* from different regions can also have different oil and oil components, for example between traditional and non-traditional producing areas, because different environmental conditions can cause changes in the active ingredients and alter the regulation of transcription genes that control the formation of secondary metabolites. As a result, the same species variety, *C. wenyujin*, can produce rhizomes with different qualities depending on the region and conditions in which it was cultivated; this can cause serious problems in quality control.

A notable characteristic of plants is their ability to respond plastically to unfavorable environmental pressures [20,21]. Plants display a series of delicate biochemical plasticity responses to environmental stress involving many aspects such as development, evolution, molecular biology, anatomy, physiology, genetics, and biochemistry [22,23]. These mechanisms are crucial for predicting changes to community composition, distribution, and crop productivity

under continuously changing environments [24, 25].

Environmental factors have a strong relationship with the formation of organic medicinal materials. Various environmental factors can affect and even stress medicinal plants during growth, influencing the formation and accumulation of terpenoids, ketones, phenols, alkaloids, volatile oils, and other secondary metabolite substances. These stress factors may include biological factors like herbivores, pests, microorganisms, and diseases, and abiotic factors such as water, temperature, light, and soil [26,27]. For instance, the oil content of *C. Wenyujin* rhizomes cultivated on lime soil is higher than that of rhizomes cultivated on acidic red soil, indicating that the oil content of Rhizoma Curcuma can be affected by factors such as soil quality and climate, the chemical and physical properties of soils differ based on the parent material, thus altering the growth conditions and quality of the plant [28].

The authenticity of botanical medicinal herbs is determined by the formation and accumulation of specific secondary substances. Isopentenyl pyrophosphate (IPP) is an important intermediate in the biosynthesis pathway of plants for the formation of terpenoids and steroids under the action of a series of enzymes; steroidal alkaloids can also be formed through other pathways. IPP is formed as a common intermediate in both the mevalonate pathway (MVA) and 2-methyl-D-erythritol-4-phosphoric acid pathway (MEP); thus, IPP can pass through cell walls to move between these two pathways [29].

Abiotic environmental factors (such as light, temperature, moisture, and nutrition) affect metabolism and the synthesis of metabolites predominantly by influencing gene expression and transcriptional factors in the metabolic pathways of secondary metabolites [30]. For example, the

main mechanism of action of abiotic environmental factors on plant flavonoids is to influence the expression of key genes in flavonoid metabolic pathways; the regulation of these key genes subsequently impacts other related genes in this pathway. Studies have demonstrated that UV-B radiation induces an increase in the flavonoid production of plants [31]. Lillo et al. (2008) found that the expression of the flavonoid metabolism-related genes (4CL, CHI, F3H, DFR, ANS, FLS1, AGT/FGT, AAT1, and GST) in *Arabidopsis* seedlings was significantly enhanced under intense light conditions [32]. Strong light can induce an increase in PAP1 and PAP2 transcription levels in *Arabidopsis thaliana*, thereby promoting flavonoid synthesis and accumulation [33, 34]. Koyama et al. (2012) demonstrated that shade treatment can significantly reduce the concentration of flavonols in cabernet sauvignon grapes [35]. Zhang et al. (2016) found that the expression of genes related to flavonoid metabolism (PAL, F3H, CHS, 4CL, FLS and C4H) was up-regulated at low temperatures [36]. Additionally, short-term drought stress is conducive to the synthesis and accumulation of flavonoids in *Scutellaria* and the increase of enzyme activities such as PAL and C4H [37]. Key enzymes in the synthesis and accumulation of flavonoids are also affected by varying degrees by the status of nutrients in the soil [38]. Furthermore, synthesis and secondary metabolite accumulation are often not controlled by single factors; in most cases, they are affected by a variety of environmental factors or several environmental factors working together. The subtropical monsoon climate in Wenzhou and the tropical monsoon climate in Haikou have many different natural environmental conditions, which may cause large differences in the secondary metabolites of *C. Wenyujin* rhizomes sourced from the two regions.

In recent years, drug companies from Haikou, Hainan Province ~~(such as Haikou Bikai~~

~~Pharmaceutical Co., Ltd.)~~ have introduced the local cultivation of *C. wenyujin* from plant material originally sourced from Ruian, Wenzhou, Zhejiang Province. This Haiku cultivation is for the production of medicines related to *Curcuma* and aims to meet the growing demand for *C. wenyujin* materials (volatile oil, crude extracts, and raw materials). In this study, the main substances and genetic characteristics of rhizomes of *C. wenyujin* sourced from Wenzhou and Haikou were determined to assure the quality of *C. wenyujin* rhizomes. The main objective was to analyze the constituents of essential oils such as terpene, curcumin, polysaccharide, starch, and other important substances from *C. Wenyujin* rhizomes from the traditional (Wenzhou) and introduced (Haikou) production areas. There have been some transcriptome studies of the ginger family (*Curcuma zedoaria* (Christm.) Roscoe) [39-41]; however, to date, the genetic variation of metabolic pathways in different *C. wenyujin* producing areas has not been reported. Currently, the transcriptome of *C. wenyujin* from traditional and introduced production areas and the mechanisms responsible for the interspecific differences in primary and secondary metabolite accumulations from *C. Wenyujin* rhizomes between traditional and introduced production areas have not been well-studied. Thus, in this study, the transcript profiles of *C. Wenyujin* rhizomes produced in traditional (Wenzhou) and introduced (Haikou) areas were investigated using Illumina and RNA-sequencing (RNA-seq), and the variations in the main components of the volatile oil terpene, curcumin, polysaccharide, and starch substances of Rhizoma Curcumae, and the related genes in the corresponding pathways of *C. wenyujin*, were investigated. This provided the opportunity to evaluate the quality of the plant and expound the molecular mechanisms in the biosynthetic pathways of *C. wenyujin* related substances between these two producing areas, and

provide the base for the determination of the medicinal value, development, and utilization of Rhizoma Curcumae wenyujin in Haikou.

Materials and methods

Plant materials and treatment conditions

The traditional (Wenzhou) *C. wenyujin* samples were purchased from Taoshan Town, Ruian, in Zhejiang Province on December 5, 2017. The introduced (Haiku) *C. Wenyujin* samples were obtained from the introduced cultivation fields at the Haikou branch of the Institute of Medicinal Plants on December 5, 2017. The rhizomes of the *C. wenyujin* plants were selected as the experimental material. The *Curcuma* samples were identified as *Curcuma wenyujin* Y.H. Chen & C. Ling. by Li-Rongtao, Associate Professor from the Institute of Medicinal Plants, Chinese Academy of Medical Sciences.

RNA isolation, quantification, and qualification

1% agarose gels were used to examine the contamination and degradation of RNA. The purity was examined with a Nano spectrophotometer (Photometer®, IMPLEN, CA, USA), the concentration was determined by a Qubit®2.0 Fluorometer Kit (RNA Assay, Life Technologies, CA, USA), and an Agilent 2100 Bioanalyzer system (Nano 6000, Agilent Technologies, CA, USA) was used to evaluate the RNA integrity.

Library preparation and sequencing

An RNA library was prepared using a total of 3 μg RNA per sample, which was analyzed using high throughput Illumina sequencing technology at the Beijing BioMarker Technologies Company. According to the manufacturer's recommendation, an RNA Library Prep Kit (NEB for Illumina, USA) was used for generating the sequencing library and the sequences of each sample were attributed by using index codes. mRNA was extracted from the total RNA using Poly-T oligo-attached magnetic beads. Fragmentation was achieved by crushing the sample with divalent cations in NebNext first-chain synthetic buffer (5X) at an elevated temperature. The first chain cDNA synthesis was performed using RNase-H (Invitrogen). Polymerase I (Invitrogen) and RNase-H (Invitrogen) were used for the second chain cDNA synthesis. The remaining overhangs were transformed into blunt ends by using polymerase or exonuclease reactions. After the adenylation of the DNA fragments (3′ ends), the junction (NEBNext), with a hairpin loop structure, was ligated in order to prepare for hybridization. To preferentially select cDNA fragments of 150-200 bp length, an XP system (AMP, Beckman Coulter, Beverly, USA) was applied to separate the DNA fragments. Prior to PCR, 3 μL USER enzyme (NEB, USA) was added to the samples containing the size selected adaptor-ligated cDNA and samples were heated for 37°C for 15 min, followed by 95°C for 5 min. PCR was then carried out using DNA polymerase (Phusion High-Fidelity), Index (X) and Universal Primers (PCR), the XP system (AMP, Beckman Coulter, Beverly, USA) was applied to purify the PCR products, and the library quality was evaluated using an Agilent 2100 Bioanalyzer system (Nano 6000, Agilent Technologies, CA, USA).

Data analysis and assembly

The internal Perl script was first used to handle the raw read data in fastq (fq) format. Clean reads were acquired by deleting reads including adapters, poly-N and low-quality reads from the raw data. Meanwhile, the sequence repetition, GC, Q20 and Q30 levels of clean read data were statistically managed. The left read1 files in all library examples were placed in a larger file (left fq), and the right read2 files were placed in another larger file (right fq). The assembly of the transcriptome was done based on left/right fq data using the Trinity method, with min_kmer_cov set to 2 and all the other parameters automatically set to default [42,43].

Unique sequence annotation and functional classification

Unique sequence genes were annotated and functionally classified based on Clusters of Orthologous Groups of proteins (COG), KOG, eggNOG, protein sequence data libraries for manual annotation and review (Swiss Prot), Gene Ontology (GO) and the Kyoto Encyclopedia of Genes and Genomes (KEGG) [44].

Quantification of gene expression levels

RSEM was applied to analyze the levels of gene expression in each sample [45]. Clean read data were mapped onto the assembled transcriptome, and then the read counts per gene were obtained from the database.

Differential expression analysis

The DESeq method (R package, 1.10.1) was applied to analyze the differential expression of the genes. The *P* value results were regulated using the Benjamin and Hochberg method (1995) for controlling the false discovery rate (FDR), and genes at a *P value* < 0.05 were classified as the differentially expressed genes (DEGs) [44,48].

Gene Ontology (GO) enrichment analysis

The topGO R package, based on the Kolmogorov-Smirnov test, was used to analyze the enrichment of the DEGs [44, 46].

KEGG pathway enrichment analysis

KOBAS 2.0 software was applied to test the statistical enrichment of DEGs in the KEGG pathways [47,48].

Sample preparation and GC-MS conditions

Sample materials were taken from large tubers of Rhizoma Curcumae, boiled, sliced, and dried for 24 h at 50 °C.; the volatile oil was then extracted with reference to the Chinese Pharmacopoeia [1]. A Gas Chromatography-Mass Spectrometer (GC-MS) (Agilent, model: 7890A-5975C) was used for detection in the following chromatographic conditions: DB-5MS column (0.25 mm × 30 m, 0.25 μm); helium carrier gas; EI ion ionization method; 200°C source temperature; 70 eV electron

energy;, 250°C interface temperature;, 150 μA emission current; 35 to 455 mass range; and a 0.4s scan cycle. The data processing system was Xcalibur 1.2, and the map library was NISTersion 1.7. The heating program followed was: 60 °C start temperature held for 1 min; then 4 $°C·min^{-1}$ from 60 °C to 100 °C; then 2 $°C·min^{-1}$ from 100 °C to 120 °C; then 1$°C·min^{-1}$ from 120 °C to 180 °C; then the temperature was increased at 23 $°C·min^{-1}$ from 180 °C to 230 °C, and was finally held for 10 min. Other parameters were as follows: 53 kPa column pressure; 1.04 $°C·min^{-1}$ flow rate; 0.2 μL sample volume; and a split ratio of 20:1. Eugenol (20 μL) was added to a 2 mL brown gas-phase flask and 1000 μL of *n*-hexane added; the mix was then vortexed for 1 min and filtered. An Agilent 7890A gas chromatograph (GC) coupled to a 5975 C quadrupole mass spectrometer (MS) was used to obtain the total ion chromatogram of each volatile oil. The relative value (percentage) of each peak was calculated using the peak area normalization method, and after a computer search and manual analysis, the compounds were identified with reference to the literature.

qPCR analysis

qPCR analysis was performed using a PCR detection system (IQ5 Multicolor, CFX96, Bio-Rad, Bole, USA) and a Power PCR Master Mix (SYBR green) (Takara, Beijing, China). RNase-free DNase (TaKaRa, Beijing, China) was applied to extract the total RNA from the Rhizoma Curcumae samples. 15 primers (Oligod-T) and a SuperScript (III) RT kit (Invitrogen, USA) were used for the reverse transcription (RT) reaction.

Each RT reaction had 10 μL of mix reagent (2×SYBR Green Master) (TaKaRa, Beijing, China), 10 ng of sample (cDNA) and 200 nM of gene-specific primers; the total volume was 20 μL. The cycle conditions were as follows: the cycle lasted for 30 s at 95°C; then for 5 s at 95°C for 40 cycles; then for 34 s at 60°C; this program was then followed by a 55°C-85°C melting curve, which was maintained for 5 s at each temperature. Gene-specific primers were used for PCR amplification (S1 Table). Beforehand, the suitability of the oligonucleotide sequences for annealing efficiency was assessed using the primer program (PRIMER 3.0) [49]. c121677 was chosen as an internal control gene against which the average of three repetitions was normalized. The relative expression values of the target genes were calculated by comparing the target gene cycle thresholds (CTs) and the housekeeping gene (c121677) using the $2^{-\Delta\Delta CT}$ method [50,51].

Determination of curcumin content

An Agilent Extend with a C18 column (4.6 × 250 mm, 5 μm) was applied in the following chromatographic conditions: 4 % acetonitrile and glacial acetic acid solution (45:55) was the mobile phase; the flow rate was 1.0 $mL \cdot min^{-1}$; the detection wavelength was 420 nm; the column temperature was 25°C; and the volume of injection was 10 μL. The number of theoretical plates was not less than 4,000. 6.35 mg of the curcumin reference substance was accurately weighed after ensuring that it had been dried to a constant weight (CAS: 110823-201706, China National Institute

of Food and Drug Control (CNIFDC), CN), then it was placed into a 25 mL brown volumetric flask, dissolved with methanol to the volume, shaken, and filtered as a reference stock solution. 1 ml of the reference stock solution was placed into a 10 mL brown volumetric flask, methanol was added to make up the volume and shaken; this was used as a control solution (25.4 μg·mL^{-1}). 0.5 g of accurately weighed dried powder of rhizome was placed into a 50 mL conical flask and 20 mL methanol was accurately added. The mixture was sonicated for 1 h, centrifuged, and the supernatant was obtained. 15 ml of methanol was added into the supernatant and the mixture was sonicated for 40 min, filtered, and the residue washed. It was filtered again with a small amount of methanol, and the solution washed into the above extraction solution to recover the methanol at a low temperature to a suitable amount, filtered, and the volume made up to 20 mL.

Determination of polysaccharide content

This was carried out according to the methodology in the "Chinese Pharmacopoeia" (2015 version of Appendix VA) using UV-visible spectrophotometry [1].

Preparation of reference solution

An appropriate amount of anhydrous glucose reference substance (reference D-anhydroglucose (CAS: 0833-9501, CNIFDC, CN) was accurately weighed and distilled water was added to make a 0.5 mg·mL^{-1} solution.

Preparation of the standard curve

Different quantities of the reference solution (1.0, 2.0, 2.5, 3.0, 4.0, 4.5 and 5.0 mL, respectively) were precisely weighed, and added to flasks of 5 mL total volume, the volumes of each were made up to 5 mL with distilled water, then shaken. Precise 1 mL quotients of each of the above solutions were added into 10 mL stoppered test tubes, 0.25 mL of 5 % phenol added to each, and they were mixed well. Then, to each, 3.0 mL of 80 % sulfuric acid was quickly added and the solution was heated for 15 min in a water bath at 90 °C; the test tubes were then cooled for 15 min in an ice bath. Respective reagents were used for a blank treatment. UV-Vis spectrophotometry (UV1601 spectrophotometer, Shimadzu, Japan) was used to measure the absorbance at a wavelength of 490 nm. Absorbance was plotted on the ordinate and the concentration was measured. For the abscissa, a standard curve was drawn.

Preparation method for the test solution

Rhizoma Curcumae powder (2.5 g), was passed through a 60 mesh sieve, dried at 60 °C to a constant weight, accurately weighed, and extracted in the Soxhlet Extractor with 80 % ethanol reflux for 2 h, until a residue was obtained. After the solvent evaporated, 80 mL of distilled water was added to the reflux and extracted twice for 1.5 h each time. After filtration, the filtrate was taken, and the dregs were washed with an appropriate amount of distilled water. They were moved into a 200 ml volumetric flask and distilled water was added to the mark and shaken well. Precisely 1 mL of the

test solution was measured and placed into a 10 mL stoppered test tube, and 0.6 mL of 5 % phenol solution was added. The absorbance was measured at a wavelength of 490 nm uing UV-Vis spectrophotometry (UV1601 spectrophotometer, Shimadzu, Japan) and the weight of the glucose (mg) contained in the test solution was calculated according to the standard curve that was derived in the previous section.

Starch content determination - Solution preparation

Iodine-potassium iodide solution

Iodine (2.6 g) and potassium iodide (5.0 g) were added to a small beaker and dissolved with a little distilled water; the solution was then transferred to a 1000 mL volumetric flask and diluted to the volume with distilled water.

Control starch solution preparation and assay

Soluble starch, 0.5 g (Chromatographic grade, CAS: 140602, CNIFDC, CN), was added to a 250 mL beaker to which a 100 mL distilled water was added, the solution was stirred, and heated to boiling and held at that temperature for 5 min. The solution was then cooled to 25°C, and transferred to a 250 mL volumetric flask and diluted to the volume with distilled water. The iodine-potassium iodide solution was placed in a 0.8 $mg \cdot mL^{-1}$ starch standard solution for developing color and used as the reference blank to determine the absorbance at different wavelengths with a spectrophotometer (UV1601 spectrophotometer, Shimadzu, Japan). Then, the absorbance as absorption

wavelength was plotted and the maximum absorption wavelength was measured as 600 nm.

Starch extraction and determination

Rhizoma Curcumae samples were dried naturally, crushed and then ground with a grinder, passed through a 40-mesh sieve, and placed in brown jars. 1.0 g of each sample was accurately weighed and then added into a 50 mL beaker. 20 mL of distilled water was added, the solution was stirred, heated to boiling for 5 min, and then filtered. The residue was washed with hot distilled water (5 mL × 3) and the starch was washed with an iodine-potassium iodide solution. The residue was discarded, and the filtrates were combined. The filtrate was heated to above 100°C, kept at this temperature for 10 min, and then cooled to 25°C. The filtrate was then transferred to a 50 mL volumetric flask and diluted to the volume with distilled water. Rhizoma Curcumae starch sample solution (10 mL) was added into 0.5 mL of the iodine-potassium iodide solution. The blank (10 mL distilling water + 0.5 mL iodine-potassium iodide solution) and starch solutions of different concentrations are prepared as described above, and the absorbance of the sample solutions was determined at 600 nm wavelength. The starch contents of Rhizoma Curcumae were calculated according to the standard curve.

Statistical analysis

Through three repeated measurements, the average values of each parameter were

calculated, and the standard error of the average values were obtained. Univariate analysis of variance was applied to determine the significance of the results among different treatments. Multidimensional test at a P value < 0.05 and SPSS V.13 were used for statistical analysis.

Results

Substance and volatile oil composition analysis

Curcuma wenyujin from the different regions, its rhizome (Ezhu) structure, volatile oil color, and contents were different. Volatile oil content of Rhizoma Curcuma Wehyujin from Wenzhou was greater than that in rhizomes from Haikou, and the color of the oil was bright yellow from the Wenzhou rhizomes, but dark yellow from the Haikou rhizomes (Fig 1). The method of periodic acid-schiff (PAS) reaction was combined with paraffin sectioning to show the location and quantity of starch granules in the plant tissue cells (Fig 2). The contents of oil (2.05 %), curcumin (1.46 %), and polysaccharides (8.90 %) of Rhizoma Curcuma Wehyujin from Wenzhou were significantly higher than those in rhizomes from Haikou (1.60 %, 0.91 %, and 6.15 %, respectively), and the starch content of Rhizoma Curcuma Wehyujin from Wenzhou (17.0 %) was significantly lower than that of rhizomes from Haikou (23.8 %) (Fig 3). The volatile oil composition from the different regions was significantly different; the curzerene and curdione content especially were higher in Wenzhou than those in Haikou (S2 Table). Curzerene (32.34 %), curdione (21.35 %),

and germacrene B (9.39 %) were the major components of essential oil in the Rhizoma Curcuma Wehyujin from Wenzhou of Zhejiang province. However, the main constituents of the essential oil in the rhizomes of *C. wenyujin* harvested from Haikou were curzerene (20.13 %), curdione (14.73 %), and cineole (9.76 %). Thus, the chemical constituents of the essential oils in rhizomes of *C. wenyujin* between Wenzhou and Haikou were quite different.

Fig 1. *Curcuma* rhizome and volatile oil photograph from Wenzhou and Haikou.

Fig 2. Starch (potassium iodide staining) (a,b) and oil cell (PAS reaction) (c,d) pictures of the paraffin section of *Curcuma* rhizome from Wenzhou (b, d) and Haikou (a,c) observed under a biological microscope (a, b scale=50 µm; c, d scale=100 µm).

Fig 3. The contents of oil, curcumin, polysaccharide, and starch of *Curcuma* rhizome produced in Wenzhou and Haikou.

3.2 mRNA sequencing, assembly, functional annotation and classification

The mRNA-seq libraries from *C. wenyujin* from the Haikou (introduced cultivation)

(HK) and Wenzhou (traditional cultivation) (WZ) production areas were constructed and sequenced in order to analyze the dynamics of the mRNA expression of *C. wenyujin* from the different areas. A total of six samples were sequenced for transcriptomes, yielding a total of 41.74 Gb clean data. Clean data of each sample reached up to 6.37 Gb, with Q30 percentages (a Q value of 30 corresponds to 1/1000 incorrect base rate) of more than 89.15 % in the mRNA-seq libraries of the six samples (S3 Table). 185,006 transcripts, representing 99,942 unigenes were acquired. The N50s was 1,796 bp in the transcripts, and 1,406 bp in the unigenes, and 28,375 unigenes were larger than 1 kb (S4 Table, S5 Table). BLASTX (E values < 1.00e-05) searched all unique sequences in public databases [52] and functional annotation was subsequently implemented based on databases such as NR, Swiss-Prot, COG, GO and KEGG; 51,609 unigenes (51.6%) were acquired, 48333 unique sequences (48.4%) could not be determined by sequence similarity analysis (S6 Table, S1 Fig).

The functions of the unique sequences extracted from *C. wenyujin* were classified based on the three GO terms: cellular component (CC), biological process (BP), and molecular function (MF). 15151 unigenes and 924 DEGs were classified as CC, 19906 unigenes and 1276 DEGs were classified as BP, and 21535 unigenes and 1456 DEGs were classified as MF in WZ compared to HK. The DEGs were mainly assigned to ‘cell, go: 0005623’, ‘cell part, go: 0044464’, ‘organelle, go: 0043226’, ‘membrane, go: 0016020’,‘cellular process, go: 0009987’, ‘metabolic process, go: 0008152’, ‘single-organism process, go: 0044699’, ‘binding, go: 0005488’, and

‘catalytic activity, go: 0003824’ (Fig 4). The majority of the GO terms used in the study are in accordance with GO categories defined previously [51,53].

Fig 4. Gene ontology (GO) histogram of classification in annotated unigenes WZ (Wenzhou) versus HK (Haikou). The red, green, and blue histograms represent the differentially expressed genes (DEGs) in each subcategory; the light red, green, and blue histograms represent the unigenes annotated. The right Y-axis represents the number of genes annotated or DEGs in the main categories. The left Y-axis represents the percentage of unigenes annotated or DEGs in the main categories.

1597 unique sequences were identified from the KEGG pathways (Fig 5). The enriched pathways appeared to mostly include the following pathways: endocytosis (36, 3.5 %) and phagosome (35, 3.4 %) in cellular processes; endoplasmic reticulum protein processing (70, 7.09 %), spliceosome (77, 8.0 %), ribosome (89, 9.09 %), and RNA transport (57, 5.5 %) in genetic information processing; and carbon metabolism (72, 7.12 %) and biosynthesis of amino acids (58, 5.6 %) in metabolism.

Fig 5. All unique sequences (1597) were in the KEGG classifications.

Differential gene expression

The analysis method of FPKM (fragments per kilobase of exon per million reads mapped) was applied to calculate the expression level of genes [54]. DESeq was used for DGE analysis, with the FDR set to less than 0.01. DGE analysis revealed 4620 genes that were significantly differentially expressed in WZ compared to that in HK, of which 3978 were up-regulated and 642 were down-regulated (Fig 6).

Fig 6. Differential expression of the unique sequences between WZ (Wenzhou) and HK (Haikou). Red and green points reveal significant differences in expression (FDR < 0.01). Green and red dots indicate genes with significantly different expression; green indicates down-regulated genes; red indicates up-regulated genes; black indicates genes with no significantly different expression.

To understand the differences and similarities in transcriptional changes of *C. Wenyujin* genes in WZ compared to HK, a hierarchical cluster was constructed, this analysis showed different expression patterns of the genes from *C. Wenyujin* in WZ compared to HK (Fig 7). A subset of transcripts for both clusters is presented in S7 Table, in which several transcripts are presented with their respective fold changes and false discovery rate-corrected *p*-values. Among these, genes related to terpene, curcumin, and polysaccharide pathway were up-regulated in Rhizoma Curcuma from Wenzhou, while the genes associated with starch and sugar pathways were

up-regulated in Rhizoma Curcuma from Haikou.

Fig 7. Cluster analysis of DEGs (3720) between WZ (Wenzhou) and HK (Haikou). Red represents up-regulated genes, whereas green represents down-regulated genes. The heatmap was produced from the FPKM data (T01 = HK1, T02 = HK2, T03 = HK3, T04 = WZ1, T05 = WZ2, T06 = WZ3).

Functional enrichment analysis of DEGs

TopGO was also used for the enriched GO terms for the three main clusters (CC, BP and MF) ($K_S < 0.05$, enrichment significance). Details of topGO enrichment of the WZ groups compared to HK are shown in S8 Table. In all DEGs, plastid, chloroplast (CC), methylerythritol 4-phosphate (MEP) pathway, isopentenyl diphosphate biosynthetic process, thylakoid membrane organization (BP), protein serine/threonine kinase activity, and protein kinase activity (MF) were enriched in WZ compared to HK. In up-regulated DEGs, the GO terms: pollen tube tip (CC), leaf morphogenesis (BP), polyamine oxidase activity, isomerase activity, ion channel activity, and phospholipase activity (MF) were significantly enriched in WZ compared to HK. In down-regulated DEGs, however, cell wall (CC), defence responses to fungus (BP), peroxidase activity, copper ion binding, sucrose synthase activity, heat shock protein binding, and catalytic activity (MF) were significantly enriched in WZ compared to HK (S2 Fig).

A comparison of DEGs in 26 COG classifications between HK and WZ is shown in Fig 8. In WZ, compared to HK, DEGs can be divided into the following eight categories: R (28.33 %), K (13.55 %), L (12.25 %), T (11.19 %), J (11.13 %), O (10.48 %), E (9.01 %), G 8.24 %). Therefore, general function prediction only (R), transcription (K), replication, recombination and repair (L), signal transduction mechanisms (T), translation ribosomal structure and biogenesis (J), posttranslational modification protein turnover chaperones (O), amino acid transport and metabolism (E), and carbohydrate transport and metabolism (G) play a vital role in *C. wenyujin* from the Wenzhou traditional production area.

Fig 8. COG (Clusters of Orthologous Group) functional classification of consensus sequence of DEGs (differentially expressed genes) in WZ (Wenzhou) versus HK (Haikou).

Analysis of the DEG annotation results from the KEGG enrichment pathway classifications revealed that 179 enrichment pathways in all DEGs had different levels of expression in WZ compared to HK: 117 pathways with up-regulated DEGs and 62 pathways with down-regulated DEGs (S9 Table). The significance of the enrichment factor and Q value was analyzed; the first 20 minimal Q value pathways are shown in Fig 9. 980 DEGs were assigned to KEGG pathways; 844 were up-regulated and 136

were down-regulated. The dominant and significant enrichment pathways are listed in S9 Table. All DEGs from the KEGG pathways involved in ribosome, spliceosome, endoplasmic reticulum protein processing, ubiquitin mediated proteolysis, RNA transport, phagosome, glyoxylate and dicarboxylate metabolism, and glutathione metabolism were predominantly enriched pathways. The up-regulated DEGs included spliceosome, carbon metabolism, ubiquitin mediated proteolysis, proteasome, citrate cycle (TCA cycle), aminoacyl-tRNA biosynthesis, arginine and proline metabolism, and glutathione metabolism were predominantly enriched, and the down-regulated DEGs containing ribosome, protein export, riboflavin metabolism, endoplasmic reticulum protein processing, plant hormone signal transduction, starch and sucrose metabolism were significantly enriched.

Fig 9. Enrichment of KEGG pathways in WZ (Wenzhou) versus HK (Haikou). Pathway name and enrichment intensity are shown in the right legend. The Q value is the corrected *P*-value (FDR). The rich factor is the ratio of the number of DEGs in a pathway to the number of all genes in that pathway. (a, all DEGs; b, up-regulated DEGs; c, down-regulated DEGs)

Verification of differentially expressed genes (DEGs)

To prove the reliability of the FPKM (fragments per kilobase of exon per million

reads mapped), analysis of 15 of the transcriptomes of differentially expressed genes that are involved in terpene, curcumin, polysaccharides, and starch pathways, were selected for qRT-PCR analysis. The overall correlation coefficient of the linear regression analysis between FPKM and qRT-PCR was 0.959 (R=0.959), indicating a good correlation between the transcription abundance detected by RT-PCR and the transcription spectrum of RNA sequence data analysis (S3 Fig). The results showed that the genes involved in terpene (c118842.grraph_co, c136079.grraph_co, c140218.grraph_co, c66011.grraph_co), curcumin (c135704.grraph_co, c48451.grraph_co, c89181.grraph_co), and polysaccharides (c115346.grraph_co, c115548.grraph_co, c118949.grraph_co) were significantly up-regulated in Rhizoma Curcuma Wenyujin from Wenzhou; however, a few of the genes involved in polysaccharides (c138832.grraph_co) and the genes involved in starch (c114286.grraph_co, c118416.grraph_co, c126193.grraph_co, c128492.grraph_co) pathways were significantly up-regulated in Rhizoma Curcuma Wenyujin from Haikou. These gene expression patterns were consistent with those shown in the transcriptome FPKM analysis (Fig 10).

Fig 10. qRT-PCR verification of DEGs selected in the WZ (Wenzhou) and HK (Haikou) (a), corresponding RNA-seq (b). Using C121677 as a control/reference gene, the relative expression was calculated and acquired according to $2^{-\triangle\triangle Ct}$ ratio. The asterisks (*, $P < 0.05$; **, $P < 0.01$; ***, $P < 0.001$) demonstrate significant

variations. Error bars indicate mean ± SD (n = 6).

Transcription factor analysis

Transcription factor analysis revealed that 57 classes of transcription factors had dynamic changes in Haikou compared to Wenzhou. Compared to the Haikou area, 52 types of transcription factors of Rhizoma Curcuma Wenyujin were up-regulated in Wenzhou. The expression levels of RLK, C2H2, Zn-clus, TKL, bZIP, CAMK, AP2/ERF, AGC, STE, C2C2, CMGC, C3H, SNF2, WRKY, bHLH, GNAT, MYB, HSF, and NAC were significantly up-regulated. Among them, the gene expression of some transcription factors only showed up-regulation, while few genes in RLK bZIP, AP2/ERF, WRKY, bHLH, MYB, HSF, and NAC transcription factor classes also showed down-regulation in Rhizoma Curcuma Wenyujin from WZ compared to HK (S4 Fig, S10 Table).

Discussion

Environmental factors play an important role in the growth of plants. Environmental quality and climatic conditions in different growing areas are the most important causes of variation in primary and secondary metabolites [18, 19].

Previous studies have reported large variations in the volatile oil content and its composition, curcumin, and polysaccharides among *Curcuma* species and Rhizoma

Curcumae from different areas [55-58]. Our data confirm that there are large differences in the contents of volatile oil and its composition, curcumin, polysaccharide, and starch from Rhizoma Curcuma Wenyujin between the traditional production areas in Wenzhou and the introduced production areas in Haikou. In this study, the contents of the volatile oil terpene, curcumin, and polysaccharides of Rhizoma Curcuma Wenyujin from Wenzhou were significantly higher than those in rhizomes from Haikou, but the starch content was significantly lower than that of rhizomes from Haikou. Additionally, there are certain differences in the composition of volatile oil from Rhizoma Curcuma Wenyujin between Wenzhou and Haikou. Similarly, Huang et al (2014) also found that the volatile oil content of Rhizoma Curcuma Wenyujin from Haikou (2.9 %) was lower than that of rhizomes from Zhejiang (3.0 %); they reported remarkable differences in the volatile oil constituents from different habitats [59]. Reports have also shown differences in the chemical constituents of volatile oils from *C. Wenyujin* in different planting areas, and state that the quality of medicinal materials was changed significantly [17,60,61]. According to the origin of varieties, cultivation area, cultivation techniques and extraction methods, the content of volatile oil may change [8]. Cao et al. reported that the content of volatile oil and curcumin varied greatly in different experimental areas, with curcumin content varying from 1.5% to 5% and volatile oil content varying from 0.4% to 0.7% [61].

In our study, RT-PCR and transcription RNA-seq analysis showed that the genes

involved in terpene, curcumin, and polysaccharide production were significantly up-regulated in Rhizoma Curcuma Wenyujin from Wenzhou. This suggests these genes play a significant role in the metabolite changes of *C. wenyujin* between different areas. However, a few genes involved in polysaccharide pathways and most genes related to starch pathways were significantly up-regulated in Rhizoma Curcuma Wenyujin from Haikou. These results agreed with previous reports that Rhizoma Curcuma Wenyujin from Wenzhou had higher contents of the volatile oil, terpene, curcumin, and polysaccharide and lower starch contents than those in rhizomes from Haikou; the gene expression patterns were also consistent with those in the transcriptome FPKM analysis (Fig 3, Fig 10). Huang et al. (2013) showed that the reason for the difference in starch content between different producing areas is not the influence of latitude and longitude, but may be related to local soil, temperature, light, and cultivation measures [62]. Starch formation is a dark reaction through photosynthesis; the essence of the dark reaction is a series of enzymatic reactions in the chloroplast matrix, and the influencing factors are temperature, CO_2 concentration, pH, etc. It can therefore be speculated that differences in these environmental factors between Wenzhou and Haikou caused differences in the content of starch or other substances in Rhizoma Curcuma Wenyujin. Gene expression analysis revealed that curcumin contents were greatly affected by environmental factors and nutritional changes [19].

Transcriptomics previously analyzed and studied gene expression levels and diversity

profiles of different tissues in insects, animals, and plants [63-65]. Studying the biosynthesis of special metabolites from non-model medicinal plants is an important goal of transcriptomics [66, 67].

In this study, hierarchical clustering was used to obtain a complete transcriptional profile of *C. Wenyujin* from the Wenzhou and Haikou production areas. Results indicated that Rhizoma Curcuma Wenyujin from Wenzhou had significant differences in the transcriptional profiles of the co-expressed transcripts when compared to rhizomes from Haikou. Among these co-expressed transcripts, the number of genes up-regulated was greater than the number of of genes down-regulated in Wenzhou, when compared to those in Haikou. The KEGG enriched pathway appeared mainly in the pathways including endocytosis and phagosome in cellular processes; ribosome, endoplasmic reticulum protein processing, RNA-transport and spliceosome in genetic information processing; and carbon metabolism and biosynthesis of amino acids in metabolism. These results indicate that the genes regulating endocytosis and phagosome, protein processing, spliceosome, ribosome, RNA transport, and carbon and amino acids in metabolism and biosynthesis pathways show significant differential expression in Rhizoma Curcuma Wenyujin from Wenzhou compared to Haikou.

In our study, 980 DEGs were allocated to KEGG pathways. The DEGs of the KEGG pathways involved in spliceosome, endoplasmic reticulum protein processing, ribosome, ubiquitin mediated proteolysis, RNA transport, phagosome, glyoxylate and

dicarboxylate metabolism, glutathione metabolism were predominantly enriched pathways. The up-regulated DEGs, including spliceosome, carbon metabolism, ubiquitin mediated proteolysis, proteasome, citrate cycle (TCA cycle), aminoacyl-tRNA biosynthesis, arginine and proline metabolism, glutathione metabolism, were predominantly enriched, and the down-regulated DEGs containing ribosome, starch and sucrose metabolism, endoplasmic reticulum protein processing, plant hormone signal transduction, protein export, riboflavin metabolism were significantly enriched; this shows that both secondary metabolites and primary metabolites have been actively biosynthesized. However, plant hormone signal transduction, starch and sucrose metabolism were inhibited and down-regulated in Rhizoma Curcuma Wenyujin from Wenzhou. These annotation processes for DEGs provide a valuable resource for discerning signal transduction pathways and specific genes of Rhizoma Curcuma Wenyujin from both traditional and introduced areas [51]. This may be especially useful in providing information for examination of the biosynthesis mechanism of *C. wenyujin* (S5 Fig, S6 Fig, S7 Fig).

In our study, in all DEGs, the GO terms of plastid, chloroplast (CC), methylerythritol 4-phosphate (MEP) pathway, isopentenyl diphosphate biosynthetic process, thylakoid membrane organization (BP), protein kinase activity, and protein serine/threonine kinase activity (MF) were predominantly enriched in WZ compared to those in HK. In up-regulated DEGs, the GO terms of pollen tube tip (CC), leaf morphogenesis (BP), polyamine oxidase activity, isomerase activity, ion channel activity, and

phospholipase activity (MF) were significantly enriched in WZ compared to those in HK. However, in down-regulated DEGs, the GO terms of cell wall (CC), defense response to fungus (BP), peroxidase activity, copper ion binding, sucrose synthase activity, heat shock protein binding, and catalytic activity (MF) were significantly enriched in WZ compared to those in HK. This is because Rhizoma Curcuma Wenyujin grown under different environmental conditions needs different metabolites and energy to respond to different conditions. Therefore, these DEGs play a significant role when *C. Wenyujin* is grown in different environmental conditions.

When plants suffer abiotic stresses such as drought and elevated temperatures, plants stimulate transcription factors through a series of signaling. The stimulated transcription factors bind to the corresponding cis-acting element to initiate a particular gene expression, thereby responding to the abiotic stress. To date, the plant transcription factors known to respond to abiotic stress mainly include AP2/EREBP, MYB, WRKY, bZIP, and HSFs [68-73]. AP2/EREBP, MYB, WRKY, bZIP, HSFs, RLK, bHLH, NAC, and CAMK, and their regulation responds to a variety of biotic and abiotic pressures, plant growth and material metabolism processes, including trichome and seed coat development, embryogenesis leaf senescence, biosynthesis pathways, and regulation of hormonal signals [74-78]. C2H2 and C2HC zinc finger structures belong to the WRKY family transcription factor from which the WRKY family can be explained [71]. In this study, the expression levels of RLK, C2H2, Zn-clus, TKL, bZIP, CAMK, AP2/ERF, AGC, STE, C2C2, CMGC, C3H, SNF2,

WRKY, bHLH, GNAT, MYB, HSF, and NAC were significantly up-regulated; thus, the genes of transcription factors known to be reactive to abiotic environments were significantly up-regulated. It can be speculated that the change in composition and gene regulation for Rhizoma Curcuma Wenyujin between the traditional (Wenzhou) and introduced (Haikou) production areas is likely to be caused by differences in environmental factors between the two regions.

Conclusions

In our study, we focused on changes in the transcriptome and gene expression of pathways related to differences in oil terpene, curcumin, polysaccharide, and starch of *Curcuma wenyujin* in traditional (Wenzhou) and introduced (Haikou) producing areas. Our findings suggest that the composition of volatile oil, curcumin, and polysaccharide in rhizomes from Wenzhou is higher and the starch content is lower than those in rhizomes from Haikou. Oil components significantly varied between Wenzhou and Haikou rhizomes, and there were significant differences in the volatile oil composition of *C. wenyujin* between the two areas. The transcriptome analysis showed that the transcriptional genes between Wenzhou and Haikou rhizomes are very different. Compared to Haikou rhizomes, the expression of volatile oil, curcumin, and polysaccharide pathway-related genes were significantly up-regulated in Wenzhou rhizomes, but starch-associated genes were significantly down-regulated. The expression of transcription factors (such as RLK, C2H2, bZIP, CAMK, AP2/ERF,

WRKY, bHLH, MYB, HSF, and NAC) usually associated with abiotic environmental stress were significantly up-regulated. This study further provides an insight into the molecular mechanisms of *C. wenyujin* due to environmental differences between these two regions. The study also presents new methodologies that may be useful for testing the authenticity of Chinese herbal medicines.

Acknowledgements

We would like to thank Editage (www.editage.cn) for English language editing of . this manuscript.

Author Contributions

Conceptualization: Lilan Lu.

Data curation: Lilan Lu, Peiwei Liu.

Supervision: Yuxiu Zhang, Caixia Wang, Jian Feng.

Validation: Peiwei Liu.

Writing – original draft: Lilan Lu.

Writing – review & editing: Jianhe Wei.

References

1. Committee, CP. Pharmacopoeia of the People's Republic of China. Vol. I. ed. China Chemical Industry Press: Beijing. 2015; pp, 234-239.
2. Nie XH, Ao ZH, Yin GY, TaoWY. Effect of Extraction Techniqueson the Components and Antitumor Activity in Vitro of Volatile Oil from *Curcuma Wenyujin.* Pharmaceutical Biotechnology. 2003; 10: 152-154.
3. Li W, Hong B, Li Z, Li Q, Bi K. GC-MS method for determination and pharmacokinetic study of seven volatile constituents in rat plasma after oral administration of the essential oil of *Rhizoma Curcumae*. Journal of Pharmaceutical & Biomedical Analysis. 2017; 149: 577-585. http://doi.10.1016/j.jpba.2017.11.058 PMID: 29197300
4. Lobo R, Prabhu KS, Shirwaikar DA, Shirwaikar A. *Curcuma zedoaria Rosc.* (white turmeric): a review of its chemical, pharmacological and ethnomedicinal properties. J Pharm Pharmacol. 2009; 61(1): 13-21. http://doi. 10.1211/jpp/61.01.0003 PMID: 19126292
5. Dang YY, Li XC, Zhang QW, Li SP, Wang YT. Preparative isolation and purification of six volatile compounds from essential oil of *Curcuma wenyujin* using high-performance centrifugal partition chromatography. J Sep Sci. 2010; 33(11): 1658-1664. http://doi.10.1002/jssc.200900453 PMID: 20405479
6. Zheng S, Yang H, Zhang S, Wang X, Yu L, Lu J, Li J. Initial study on naturally occurring products from traditional Chinese herbs and vegetables for chemoprevention. J Cell Biochem Suppl. 1997; 67(S27): 106-112.

http://doi.10.1002/(SICI)1097-4644(1997)27%2B<106::AID-JCB17>3.0.CO;2-L PMID: 9591200

7. Lv D, Cao Y, Dong X, Chen X, Lou Z, Chai Y. Analysis and pharmacokinetic study of curdione in *Rhizoma Curcumae* by UPLC/QTOF/MS. Biomed Chromatogr. 2014; 28(6): 782-787. http://doi.10.1002/bmc.3118 PMID: 24861744

8. Quan X, Kui JZ, Zhao GH, Ping Z, Tina TXD, Shao PL, Karl WKT. Molecular genetic and chemical assessment of Rhizoma Curcumae in China. J Agric Food Chem. 2005; 53(15): 6019-6026. http://doi. 10.1021/jf0508495 PMID: 16028990.

9. Zhou Y, Xie M, Song Y, Wang WP, Zhao HR, Tian YX, Wang Y, Bai SJ, Zhao YC, Chen XY, She GM. Two traditional Chinese medicines Curcumae radix and Curcumae rhizoma: an ethnopharmacology, phytochemistry, and pharmacology review. Evid. Based Complement. Alternat. Med. 2016; 3: 1-30. http://dx.doi.org/10.1155/2016/4973128 PMID: 27057197

10. Zhang L, Yang Z, Huang Z, Zhao M, Li P, Zhou W, Zhang K, Zheng X, Lin L, Tang J, Fang Y, Du ZY. Variation in Essential Oil and Bioactive Compounds of *Curcuma kwangsiensis* Collected from Natural Habitats. Chemistry & Biodiversity. 2017; **14**(7): e1700020. http://doi.10.1002/cbdv.201700020 PMID: 28398606

11. Zhang L, Yang Z, Wei J, Su P, Chen D, Pan W, Zhou W, Zhang K., Zheng X, Lin L, Tang J, Du ZY. Contrastive analysis of chemical composition of essential oil from twelve *Curcuma* species distributed in China. Industrial Crops & Products. 2017; **108**: 17-25.

12. Li X, Sun YT, Zhang ZQ, Hu B, Liu SC. Determination ofβ-element,curcumol,germacrone and curdione of Zedoary turmeric oil by GC. Chinese Journal of Pharmaceutical Analysis.

2009; 29: 1832-1836.

13. Mao CQ, Lu TL, Jiang GF, Su T, Cai BC, Determination of curdione, curcumol, germacrone, and β-elemene in processed *Curcumae Rhizoma* from different habitats. Chinese Traditional & Herbal Drugs. 2013; 44(3): 305-308..

14. Manzan AC, Toniolo FS, Bredow E, Povh NP. Extraction of essential oil and pigments from *Curcuma longa* L. by steam distillation and extraction with volatile solvents. J. Agric. Food Chem. 2003; 51, 6802-6807. http://doi. 10.1021/jf030161x PMID: 14582978

15. Mehla J, Reeta KH, Gupta P, Gupta YK. Protective effect of curcumin against seizures and cognitive impairment in a pentylenetetrazole-kindled epileptic rat model. Life Sci. 2010; 87(19-22): 596-603. http://doi. 10.1016/j.lfs.2010.09.006 PMID: 20840851

16. Yang FQ, Wang YT, Li SP. Simultaneous determination of 11 characteristic components in three species of *Curcuma rhizomes* using pressurized liquid extraction and high-performance liquid chromatography. Journal of Chromatography A. 2006; 1134(1): 226-231. http://doi. 10.1016/j.chroma.2006.09.048 PMID: 17018231

17. Liu ZL, Zhao NN, Liu CM, Zhou L, Du SS. Identification of insecticidal constituents of the essential oil of *Curcuma wenyujin* rhizomes active against *Liposcelis bostrychophila Badonnel.* Molecules. 2012; 17, 12049-12060. http://doi.10.3390/molecules171012049

18. Li D, Ono N, Sato T, Sugiura T, Altaf-Ul-Amin M, Ohta D, Suzuki H, Arita M, Tanaka K., Ma Z, Kanaya S. Targeted Integration of RNA-Seq and Metabolite Data to Elucidate Curcuminoid Biosynthesis in Four Curcuma Species. Plant Cell Physiol. 2015; **56**(5): 843-851. http://doi.10.1093/pcp/pcv008 PMID: 25637373

19. Inavolu SS, Sanghamitra N, Sujata M. Differential expression of *curcumin* synthase at nine agroclimatic zones as influenced by environment and nutritional variation. Journal of Biotechnology. 2016; 231: S37-S37. http://doi. 10.1016/j.jbiotec.2016.05.147

20. Givnish TJ. Ecological constraints on the evolution of plasticity in plants. Evolutionary Ecology. 2002; 16(3): 213-242. http:// doi.10.1023/A:1019676410041

21. Des Marais DL, Juenger TE. Pleiotropy, plasticity, and the evolution of plant abiotic stress tolerance. Ann N Y Acad Sci. 2010; 1206: 56-79. https://doi.10.1111/j.1749-6632.2010.05703.x PMID: 20860683

22. Sultan SE.Phenotypic plasticity for plant development, function and life history. Trends Plant Sci. 2000; 5: 537-542. http://doi. 10.1016/S1360-1385(00)01797-0 PMID: 11120476

23. Shao HB, Guo QJ, Chu LY, Zhao XN, Su ZL, Hu YC, Cheng JF. Understanding molecular mechanism of higher plant plasticity under abiotic stress. Colloids Surf B Biointerfaces. 2007; 54(1): 37-45. http://doi.org/10.1016/j.colsurfb.2006.07.002 PMID: 16914294

24. Sun CX, Li MQ, Gao XX, Liu LN, Wu XF, Zhou JH.Metabolic response of maize plants to multi-factorial abiotic stresses. Plant Biology. 2015; 18(Suppl 1): 120-9. http://doi. 10.1111/plb.12305

25. Nicotra AB, Atkin OK., Bonser SP, Davidson AM, Finnegan EJ, Mathesius U, Poot P, Purugganan MD, Richards CL, Valladares F, Kleunen M. Plant phenotypic plasticity in a changing climate. Trends Plant Sci. 2010; 15, 684-692. http://doi. 10.1016/j.tplants.2010.09.008 PMID: 20970368

26. Lin L, Li W, Shao J, Luo W, Zhao C. Modelling the effects of soil water potential on growth

and quality of cut chrysanthemum (Chrysanthemum morifolium). Scientia Horticulturae. 2011; 130(1): 275-288. https://doi.org/10.1016/j.scienta.2011.06.008

27. Mekapogu M, Sohn HB, Kim SJ, Lee YY, Park HM, Jin YI, Hong SY, Suh JT, Kwem K, Jeong JC, Kwon OK, Kim YH. Effect of Light Quality on the Expression of Glycoalkaloid Biosynthetic Genes Contributing to Steroidal Glycoalkaloid Accumulation in Potato. American Journal of Potato Research. 2016; 93(3): 264-277. https://doi.10.1007/s12230-016-9502-z

28. He XY, Cao JH, Lu MG. Experimentation of *Curcuma wenyujin* in different soil conditions. Chinese Journal of Eco-Agriculture. 2007; 15: 98-101

29. Dick CA, Buenrostro J, Butler T, Carlson ML, Kliebenstein DJ, Whittall JB. Arctic mustard flower color polymorphism controlled by petal-specific downregulation at the threshold of the anthocyanin biosynthetic pathway. PLoS One. 2011; 6(4): e18230. http://doi.10.1371/journal.pone.0018230 PMID: 21490971

30. Gershenzon J. Changes in the Levels of Plant Secondary Metabolites Under Water and Nutrient Stress. In: Timmermann, B.N., Steelink, C., Loewus, F.A. (eds) Phytochemical Adaptations to Stress. Recent Advances in Phytochemistry (Proceedings of the Phytochemical Society of North America), Springer, Boston, MA.1984; 18: 273-320

31. Winkel-Shirley B. Flavonoid Biosynthesis. A Colorful Model for Genetics, biochemistry, cell biology, and biotechnology. Plant Physiol. 2001; 126: 485-493. http://doi.10.1104/pp.126.2.485 PMID: 11402179

32. Lillo C, Lea US, Ruoff P. Nutrient depletion as a key factor for manipulating gene expression

and product formation in different branches of the flavonoid pathway. Plant Cell Environ. 2008; 31(5): 587-601. http://doi. 10.1111/j.1365-3040.2007.01748.x PMID: 18031469

33. Misson J, Raghothama KG, Jain A, Jouhet J, Block MA, Bligny R, Ortet P, Creff A, Somerville S, Rolland N, Doumas P, Nacry P, Herrerra-Estrella L, Nussaume L, Thibaud MC. A genome-wide transcriptional analysis using Arabidopsis thaliana Affymetrix gene chips determined plant responses to phosphate deprivation. Proc Natl Acad Sci USA. 2005; 102, 11934-11939. http://doi. 10.1073/pnas.0505266102 PMID: 16085708

34. Vanderauwera S, Zimmermann P, Rombauts S, Vandenabeele S, Langebartels C, Gruissem W, Inzé D, Van BF. Genome-wide analysis of hydrogen peroxide-regulated gene expression in Arabidopsis reveals a high light-induced transcriptional cluster involved in anthocyanin biosynthesis. Plant Physiol. 2005; 139(2): 806-821. http://doi. 10.1104/pp.105.065896 PMID: 16183842

35. Koyama K, Ikeda H, Poudel PR, Goto-Yamamoto N. Light quality affects flavonoid biosynthesis in young berries of Cabernet Sauvignon grape. Phytochemistry. 2012; 78: 54-64. http://doi.10.1016/j.phytochem.2012.02.026 PMID: 22455871

36. Zhang G, Feng T, Wang SS, Li WJ, Yang LY, Li JY, Ma JH, Gong M. Regulation of Different Growth Temperatures on Flavonoids Content and Their Metabolic Pathways in Tobacco Leaves. Genomics & Applied Biology. 2016; 12: 3527-3537

37. Jiang X. Effects of water factors on flavonoid metabolism of Astragalus. Jilin Agricultural University. 2013; pp, 1-125

38. Lea US, Slimestad R, Smedvig P, Lillo C. Nitrogen deficiency enhances expression of

specific MYB and bHLH transcription factors and accumulation of end products in the flavonoid pathway. Planta. 2007; 225(5): 1245-1253. http://doi.10.1007/s00425-006-0414-x PMID: 17053893

39. Annadurai RS, Neethiraj R, Jayakumar V, Damodaran AC, Rao SN, Katta MA, Gopinathan S, Sarma SP, Senthilkumar V, Niranjan V, Gopinath A, Mugasimangalam RC. De Novo Transcriptome Assembly (NGS) of Curcuma longa L. Rhizome Reveals Novel Transcripts Related to Anticancer and Antimalarial Terpenoids. PLoS ONE, 2013; 8(2): e56217. http://doi.10.1371/journal.pone.0056217. PMID:23468859

40. Prasath D, Karthika R, Habeeba NT, Suraby EJ, Rosana OB, Shaji A, Eapen SJ, Deshpande U, Anandaraj M. Comparison of the transcriptomes of ginger (*Zingiber officinale Rosc*.) and mango ginger (*Curcuma amada* Roxb.) in response to the bacterial wilt infection. PLoS One. 2014; 9(6): e99731. http://doi. 10.1371/journal.pone.0099731 PMID: 24940878

41. Sahoo A, Kar B, Sahoo S, Ray A, Nayak S. Transcriptome profiling of *Curcuma longa* L. cv. Suvarna. Genom Data. 2016; 10: 33-34. http://doi.10.1016/j.gdata.2016.09.001 PMID: 27668184

42. Grabherr MG, Haas BJ, Yassour M, Levin JZ, Thompson DA, Amit I, Adiconis X, Fan L, Raychowdhury R, Zeng QD, Chen ZH, Mauceli E, Hacohen N, Gnirke A, Rhind N, Palma F, Birren BW, Nusbaum C, Lindblad-Toh K, Friedman N, Regev A. Full-length transcriptome assembly from RNA-Seq data without a reference genome. Nat Biotechnol. 2011; 29(7): 644-652. http://doi.10.1038/nbt.1883 PMID: 21572440

43. Cheng C, Qiang L, Qiang W, Dai L, Hong Z, Jian W, Rong F. Transcriptional profiling

provides new insights into the role of nitric oxide in enhancing Ganoderma oregonense resistance to heat stress. Sci Rep. 2017; 7(1): 15694. http://doi.10.1038/s41598-017-15340-6

44. Bao J, Xing YN, Jiang HB, Li XD. Identification of immune-related genes in gills of chinese mitten crabs (eriocheir sinensis) during adaptation to air exposure stress. Fish & Shellfish Immunology.2019; 84, 885-893. http://doi.10.1016/j.fsi.2018.10.085

45. Li B and Colin ND. RSEM_ accurate transcript quantification from RNA Seq data with or without a reference genome. BMC Bioinformatics. 2011; 12: 323. http://doi.10.1186/1471-2105-12-323 PMID: 21816040

46. Alexa A and Rahnenfuhrer J. topGO Enrichment analysis for Gene Ontology. R package version 2.10.0. 2010

47. Mao X, Cai T, Olyarchuk JG, Wei L. Automated Genome Annotation and Pathway Identification Using the KEGG Orthology (KO) As a Controlled Vocabulary. Bioinformatics 2005; 21: 3787-3793. http://doi. 10.2307/1592215

48. Anders S and Huber W. Differential expression analysis for sequence count data. Genome Biol. 2010; 11: R106. https://doi.org/10.1186/gb-2010-11-10-r106

49. Rozen S and Skaletsky H. Primer3 on the WWW for General Users and for Biologist Programmers. Methods Mol. Biol. 2000; 132: 365-386. http://doi. 10.1385/1-59259-192-2:365 PMID:10547847

50. Kenneth JL, Thomas DS. Analysis of Relative Gene Expression Data Using Real-Time Quantitative PCR and the $2^{-\Delta\Delta CT}$ Method. Methods. 2001; **25**(4): 402-408. http://doi.10.1006/meth.2001.1262 PMID: 11846609

51. Hongmei L, Zhu YJ, Song JY, Xu LJ, Sun C, Zhang X, Xu YH, He L, Sun W, Xu HB, Wang B, Li XE, Li CY, Liu JY, Chen SL. Transcriptional data mining of *Salvia miltiorrhiza* in response to methyl jasmonate to examine the mechanism of bioactive compound biosynthesis and regulation. Physiol Plant. 2014; 152(2): 241-55. http://doi. 10.1111/ppl.12193. PMID: 24660670

52. Xiao R, Liang W, Yingshuai C, Guren Z. Transcriptome response to temperature stress in the wolf spider Pardosa pseudoannulata (Araneae: *Lycosidae*). Ecol Evol. 2016; 6(11): 3540-3554. http://doi. 10.1002/ece3.2142

53. Hua WP, Zhang Y, Song J, Zhao L, Wang Z. *De novo* transcriptome sequencing in *Salvia miltiorrhiza* to identify genes involved in the biosynthesis of active ingredients. Genomics. 2011; 98: 272-279. http://doi.10.1016/j.ygeno.2011.03.012 PMID: 21473906

54. Mortazavi A, Williams BA, McCue K, Schaeffer L, Wold B. Mapping and quantifying mammalian transcriptomes by RNA-Seq. Nat. Methods. 2008; 5: 621-628. http://doi. 10.1038/nmeth.1226 PMID: 18516045

55. Chand SK, Nanda S, Rout E, Mohanty JN, Mishra R, Joshi RK. De novo sequencing and characterization of defense transcriptome responsive to Pythium aphanidermatum infection in *Curcuma longa* L. Physiological and Molecular Plant Pathology. 2016; 94: 27-37. http://dx.doi.org/10.1016/j.pmpp.2016.03.008

56. Santhi R, Sheeja TE, Krishnamurthy KS. Transcriptome deep sequencing, identification of novel microRNAs and validation under drought stress in turmeric (*Curcuma longa* L.). Plant Biotechnology Reports. 2016; 10(4): 227-240. http://doi.10.1007/s11816-016-0399-2

57. Sheeja TE, Deepa K, Santhi R, Sasikumar B. Comparative Transcriptome Analysis of Two Species of Curcuma Contrasting in a High-Value Compound Curcumin: Insights into Genetic Basis and Regulation of Biosynthesis. Plant Molecular Biology Reporter. 2015; 33(6): 1825-1836. http://doi. 10.1007/s11105-015-0878-6

58. Deepa K, Sheeja TE, Rosana OB, Srinivasan V, Krishnamurthy KS, Sasikumar B. Highly conserved sequence of ClPKS11 encodes a novel polyketide synthase involved in curcumin biosynthesis in turmeric (*Curcuma longa* L.). Industrial Crops and Products. 2017; 97: 229-241. http://doi.org/10.1016/j.indcrop.2016.12.003

59. Huang X, Pan L, Wang CT. Composition analysis of essential oil of *Curcuma wenyujin* Rhizoma from Hainan Province. Hainan Medical Journal. 2014; 24(20): 3024-3027.

60. Xiang Z, Wang XQ, Gao HC, Zhang ML, Zeng S. GC-MS and HPLC metabolic profiling studies of *Curcuma wenyujin* rhizomes obtained at different harvest times. Analytical Letters. 2012; 45(1): 1-14. http://doi.10.1080/00032719.2011.565447

61. Cao J, Qi ML, ZhangY, Zhou S, ShaoQL, Fu RN. Analysis of volatile compounds in *Curcuma wenyujin* Y.H Chen et C Ling by headspace solvent microextraction-gas chromatography-mass spectrometry. Analytica Chimica Acta. 2006; 561(1): 88-95. http://doi.10.1016/j.aca.2006.01.040

62. Huang LH, Liu HL, Chen Y, Li XH, Zhang H, Zhao Y. Comparative Study of Starch Content in Ginseng from Different Regions,Years and Growth Periods. Liaoning Journal of Traditional Chinese Medicine. 2013; 6: 1206-1209.

63. Logacheva MD, Kasianov AS, Vinogradov DV, Samigullin TH, Gelfand MS, Makeev VJ,

Penin AA. De novo sequencing and characterization of floral transcriptome in two species of buckwheat (*Fagopyrum*). BMC Genomics. 2011; 12: 30. http://doi. 10.1186/1471-2164-12-30 PMID: 21232141

64. Swarbreck SM, Lindquist EA, Ackerly DD, Andersen GL. Analysis of leaf and root transcriptomes of soil-grown Avena barbata plants. Plant Cell Physiol. 2011. 52(2): 317-332. http://doi. 10.1093/pcp/pcq188 PMID: 21310848

65. Wang XW, LuanJB, Li JM, Su YL, Xia J, Liu SS. Transcriptome analysis and comparison reveal divergence between two invasive whitefly cryptic species. BMC Genomics. 2011; 12: 458. http://doi.10.1186/1471-2164-12-458 PMID: 21939539

66. Xiao M, Zhang Y, Chen X, Lee EJ, Barber CJ, Chakrabarty R, Desgagné-Penix I, Haslam TM, Kim YB, Liu E, MacNevin G, Masada-Atsumi S, Reed DW, Stout JM, Zerbe P, Zhang Y, Bohlmann J, Covello PS, De Luca V, Page JE, Ro DK, Martin VJ, Facchini PJ, Sensen CW. Transcriptome analysis based on next-generation sequencing of non-model plants producing specialized metabolites of biotechnological interest. J Biotechnol. 2013; 166(3): 122-134. http://doi. 10.1016/j.jbiotec.2013.04.004 PMID: 23602801

67. Sui C, Chen M, Xu J S, Wei J H, Jin Y, Xu YH, Sun J, Gao K, Yang CM, Zhang Z, Chen SL, Luo HM. Comparison of root transcriptomes and expressions of genes involved in main medicinal secondary metabolites from Bupleurum chinense and Bupleurum scorzonerifolium, the two Chinese official Radix bupleuri source species. Physiol Plant. 2015; **153**(2): 230-42. http://doi. 10.1111/ppl.12254. PMID: 25117935

68. Du M, Zhai Q, Deng L, Li S, Li H, Yan L, Huang Z, Wang B, Jiang H, Huang T, Li CB, Wei

J, Kang L, Li J, Li C. Closely related NAC transcription factors of tomato differentially regulate stomatal closure and reopening during pathogen attack. Plant Cell. 2014; 26(7): 3167-3184. http://doi.10.1105/tpc.114.128272 PMID: 25005917

69. Ko SS, Li MJ, Sun-Ben KM, Ho YC, Lin YJ, Chuang MH, Hsing HX, Lien YC, Yang HT, Chang HC, Chan MT. The bHLH142 Transcription Factor Coordinates with TDR1 to Modulate the Expression of EAT1 and Regulate Pollen Development in Rice. Plant Cell. 2014; 26(6): 2486-2504. http://doi.10.1105/tpc.114.126292. PMID: 24894043

70. Vermeirssen V, De Clercq I, Van Parys T, Van BF, Van de PeerY. Arabidopsis ensemble reverse-engineered gene regulatory network discloses interconnected transcription factors in oxidative stress. Plant Cell. 2014; 26(12): 4656-4679. https://doi.org/10.1105/tpc.114.131417 PMID: 25549671

71. Xu ZY, Kim SY, Hyeon DY, Kim DH, Dong T, Park YM, Jin JB, Joo SH, Kim SK, Hong JC, Hwang D, Hwang I. The Arabidopsis NAC transcription factor ANAC096 cooperates with bZIP-type transcription factors in dehydration and osmotic stress responses. Plant Cell. 2013; 25(11): 4708-4724. http://doi. 10.1105/tpc.113.119099 PMID: 24285786

72. Pautler M, Eveland AL, LaRue T, Yang F, Weeks R, Lunde C, Je B, Meeley R, Komatsu M, Vollbrecht E, Sakai H, Jackson D. FASCIATED EAR4 encodes a bZIP transcription factor that regulates shoot meristem size in maize. Plant Cell. 2015; 27(1): 104-20. http://doi. 10.1105/tpc.114.132506 PMID: 25616871

73. Mol J, Cornish E, Mason J, Koes R. Novel coloured flowers. Curr. Opin. Biotechnol. 1999; 10: 198-201. https://doi.org/10.1016/S0958-1669(99)80035-4 PMID: 10209139

74. Pandey SP and Somssich IE. The role of WRKY transcription factors in plant immunity. Plant Physiol. 2009; 150(4): 1648-1655. http://doi. 10.1104/pp.109.138990 PMID: 19420325

75. Bakshi M, Oelmüller R. WRKY transcription factor: jack of many trades in plants. Plant Signaling & Behavior. 2014; **9**(2): e27700. http://dx.doi.org/10.4161/psb.27700 PMID: 24492469

76. Rushton PJ, Somssich IE, Ringler P, Shen QJ. WRKY transcription factors. Trends Plant Sci. 2010; 15: 247-258. http://doi. 10.1016/j.tplants.2010.02.006 PMID: 20304701

77. Zhou X, Jiang Z, Yu D. WRKY22 transcription factor mediates dark-induced leaf senescence in Arabidopsis. Mol Cells. 2011; 31(4): 303-313. http://doi. 10.1007/s10059-011-0047-1 PMID: 21359674

78. Zhu Y, Functional study of maize microtubule-associated protein ZmMAP65-1a and transcription factor ZmNAC in abiotic stress. Nanjing Agricultural University. 2014; pp, 1-180.

Supporting information

S1 Table. RNA-seq verification by using RT-PCR primers in Rhizoma Curcumae.

(XLS)

S2 Table. GC-MS detection results of main volatile oil constituents in Rhizoma Curcumae from WZ (Wenzhou) and HK (Haikou).

(XLS)

S3 Table. Sample sequencing data evaluation statistics.

(XLS)

S4 Table. Assembly result statistics.

(XLS)

S5 Table. Comparison of sequencing data and assembly results.

(XLS)

S6 Table. Unigene annotation statistics.

(XLS)

S7 Table. Expression data of enriched key DEGs in different assemblies from WZ (Wenzhou) versus HK (Haikou).

(XLS)

S8 Table. Details of topGO enrichment from WZ (Wenzhou) versus HK (Haikou).

(XLS)

S9 Table. Details of KEGG enrichment from WZ (Wenzhou) versus HK (Haikou).

(XLS)

S10 Table. Transcription factor expression from WZ (Wenzhou) versus HK (Haikou).

(XLS)

S1 Fig. *Curcuma* unigene length distribution.

(TIFF)

S2 Fig. Enrichment analysis of responsive genes and transcripts using GO terms from WZ (Wenzhou) versus HK (Haikou). The node size is proportional to the number of targets in the GO category. Node color represents enriched significance; a deeper color represents higher significance (a1, a2, a3: total; b1, b2, b3: down-regulated; c1, c2, c3: up-regulated).

(PDF)

S3 Fig. Comparison of RNA-seq and qRT-PCR (quantitative real-time PCR) for 15 selected genes. log2Fold Change (WZ (Wenzhou)/HK (Haikou).

(TIFF)

S4 Fig. Transcription factor expression from WZ (Wenzhou) versus HK (Haikou).

(TIFF)

S5 Fig. Terpenoid backbone synthesis, KEGG enrichment structure from WZ (Wenzhou) versus HK (Haikou).

(TIFF)

S6 Fig. Starch and sucrose metabolism, KEGG enrichment structure from WZ (Wenzhou) versus HK (Haikou).

(TIFF)

S7 Fig. Glycolysis/ Gluconeogenesis, KEGG enrichment structure from WZ (Wenzhou) versus HK (Haikou).

(TIFF)

Figure1 Click here to download Figure Fig 1.tif

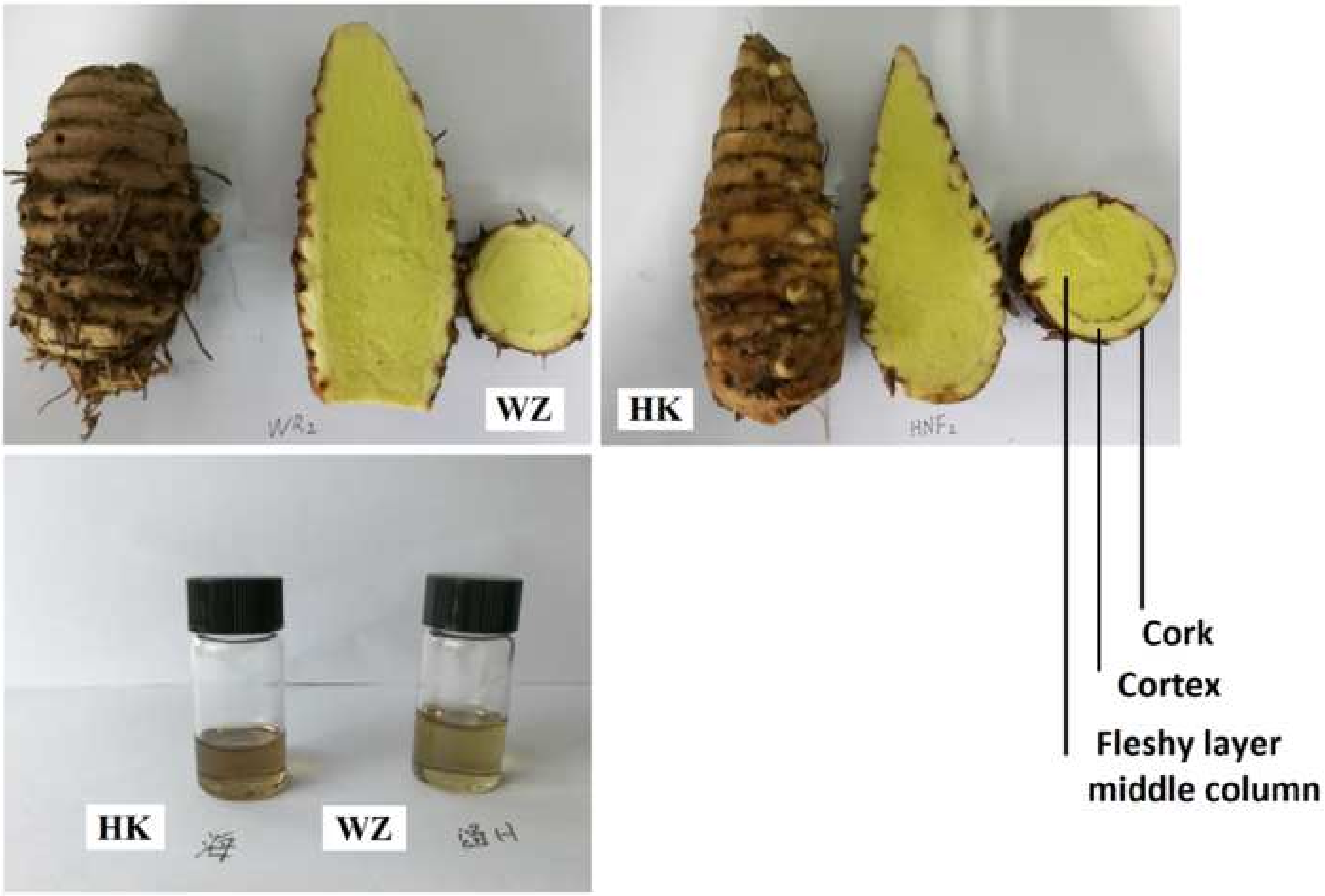

Figure2

Click here to download Figure Fig 2.tif

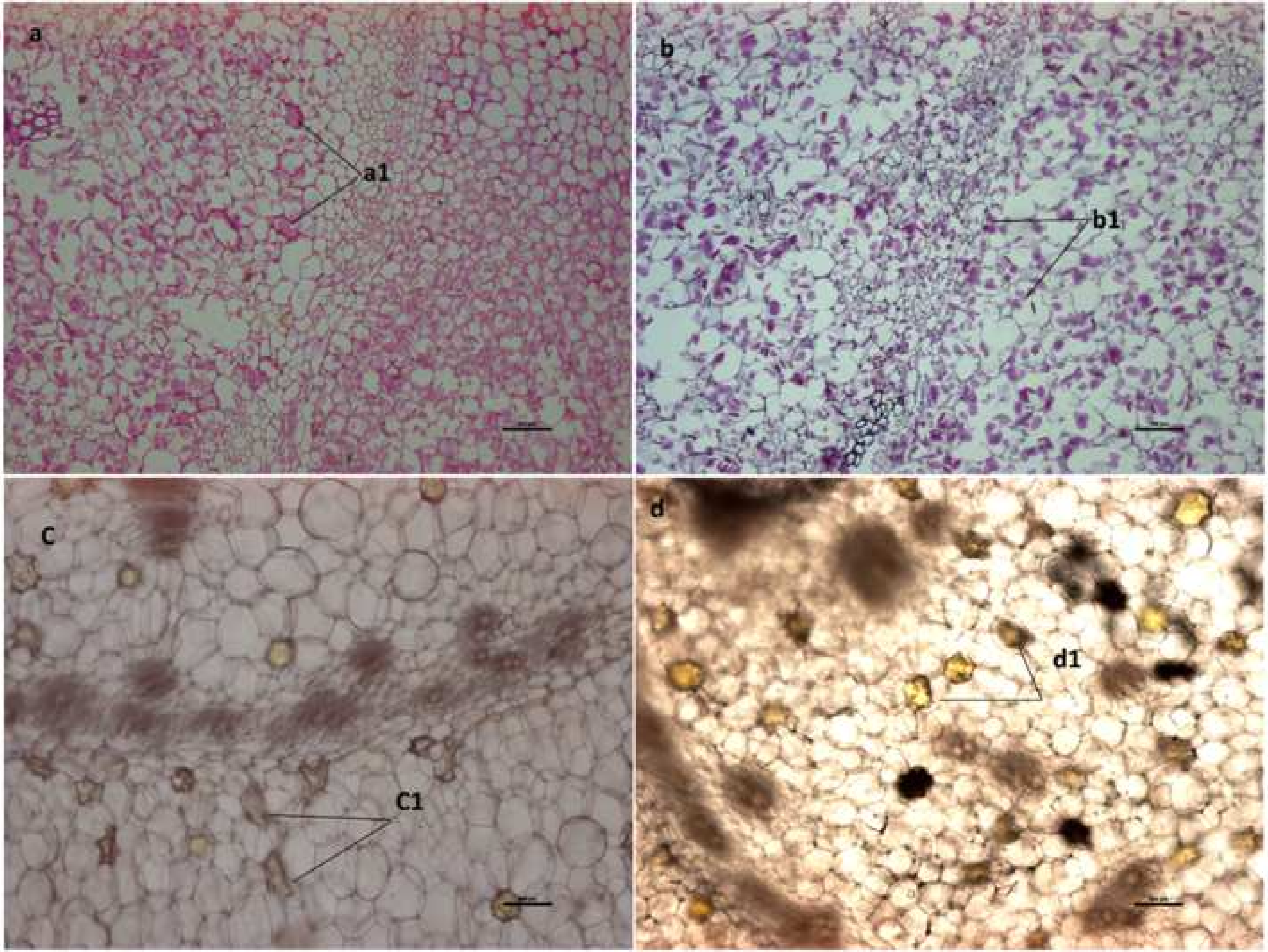

Figure3

Click here to download Figure Fig 3.tif

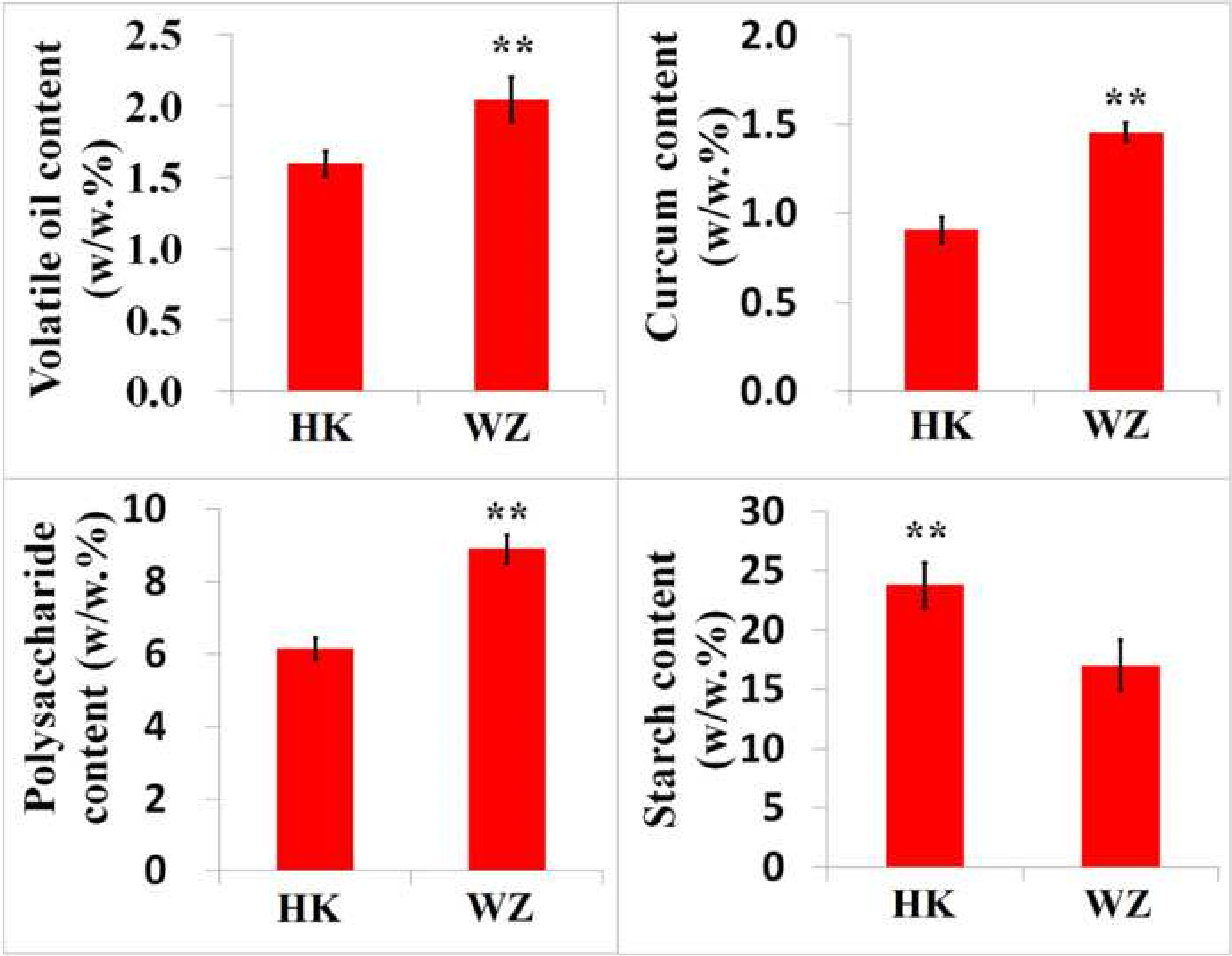

Figure4

Click here to download Figure Fig 4.tif

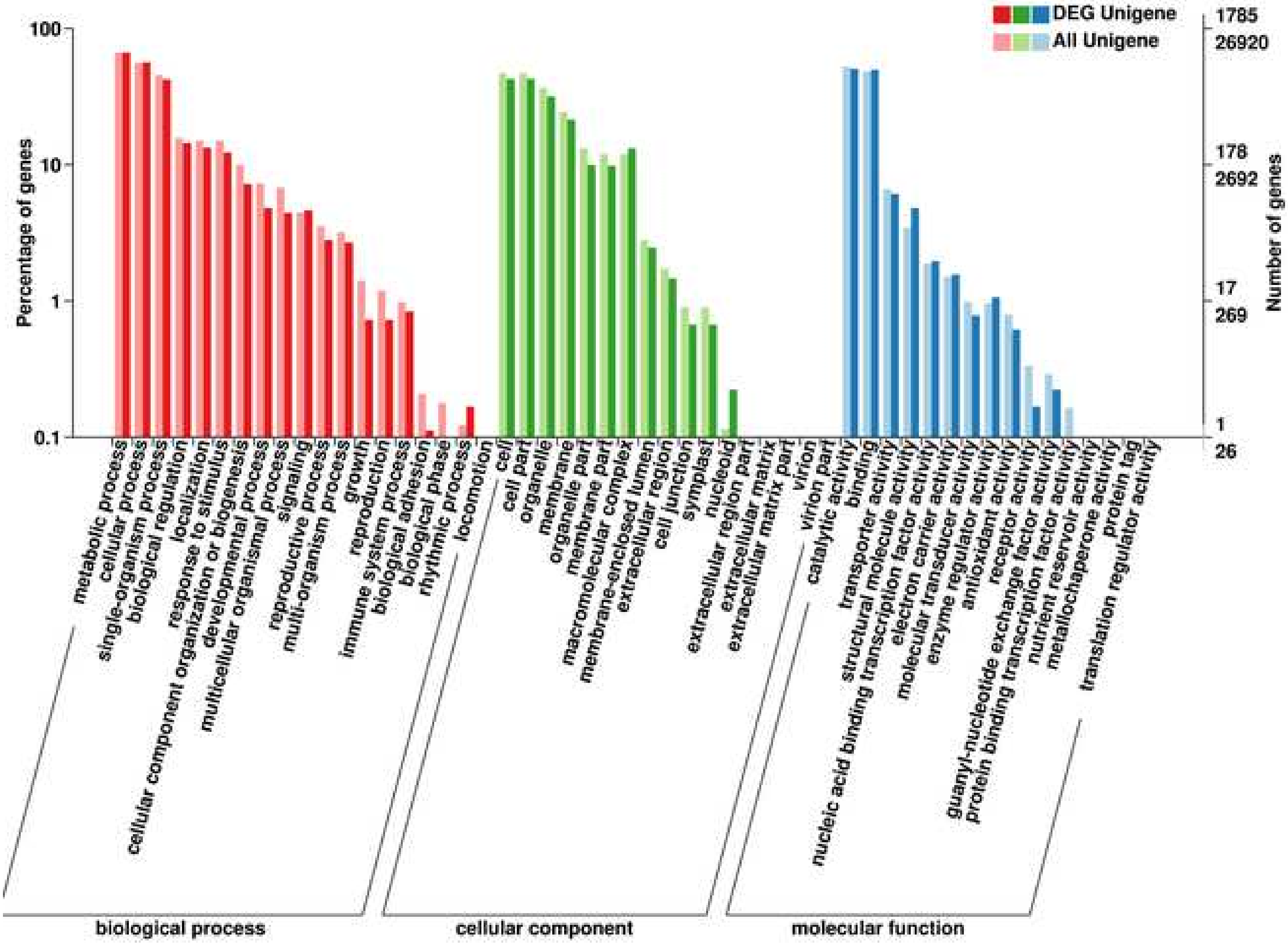

Figure5

Click here to download Figure Fig 5.tif

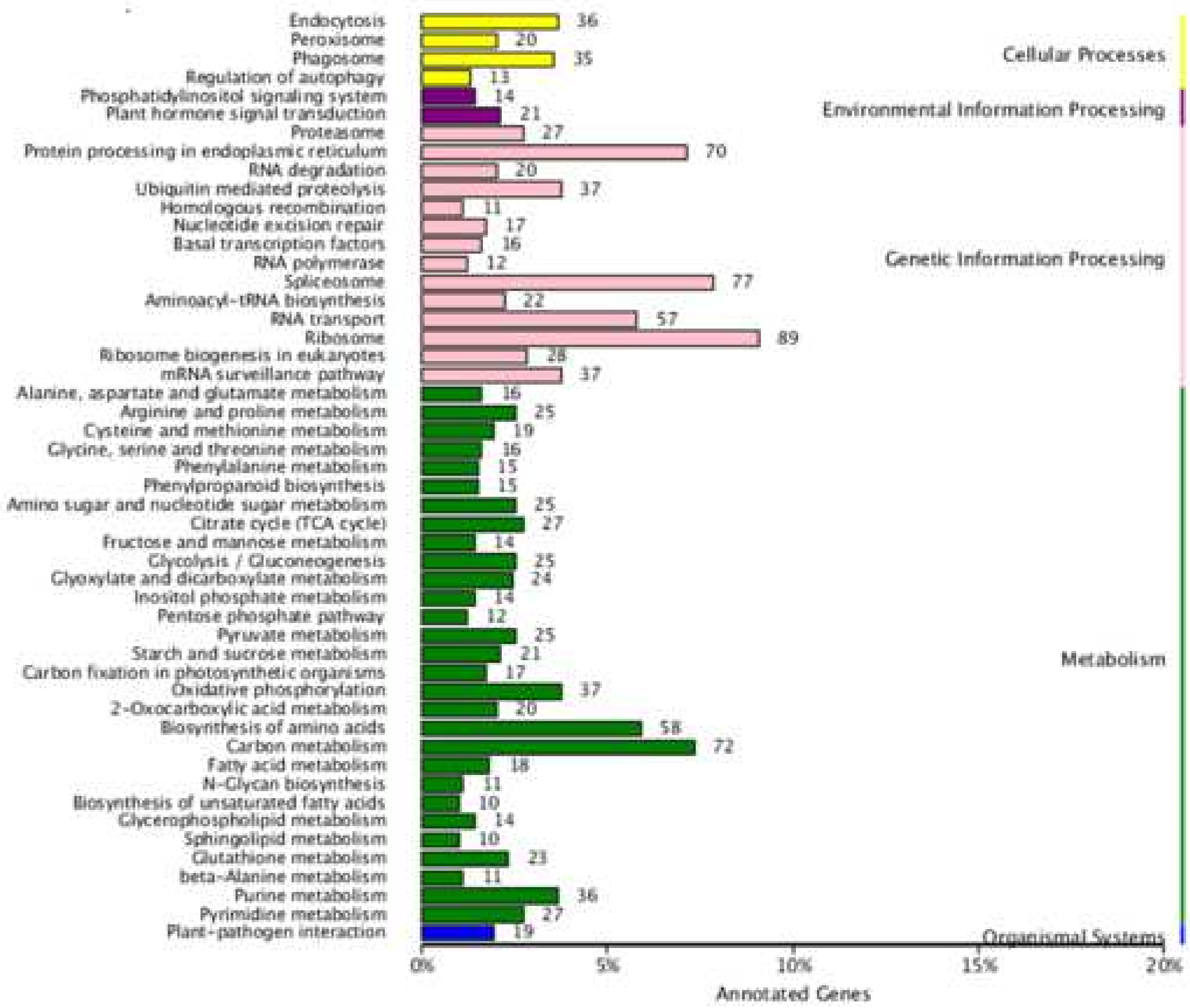

Figure6

Click here to download Figure Fig 6.tif

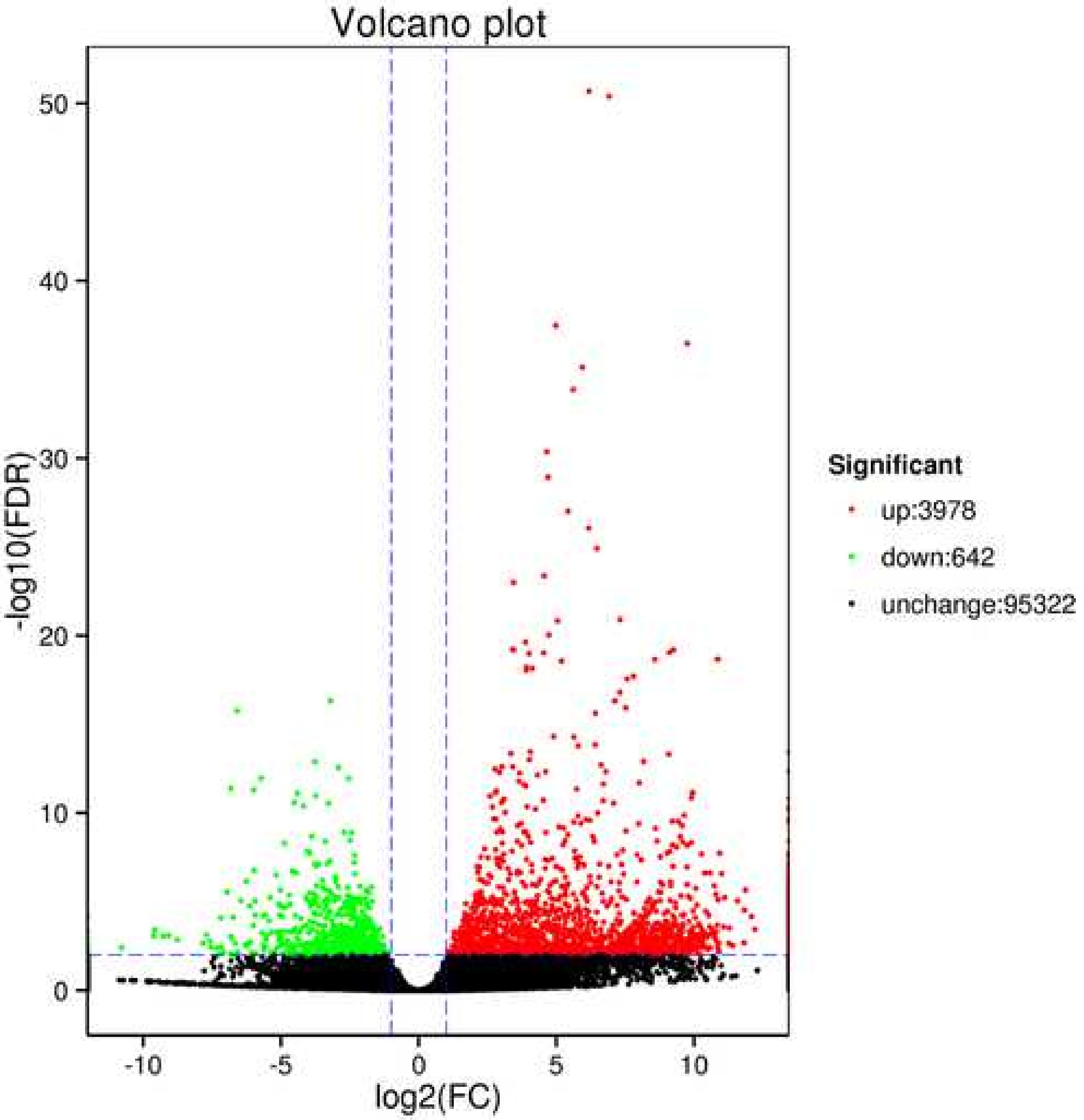

Figure7

Click here to download Figure Fig 7.tif

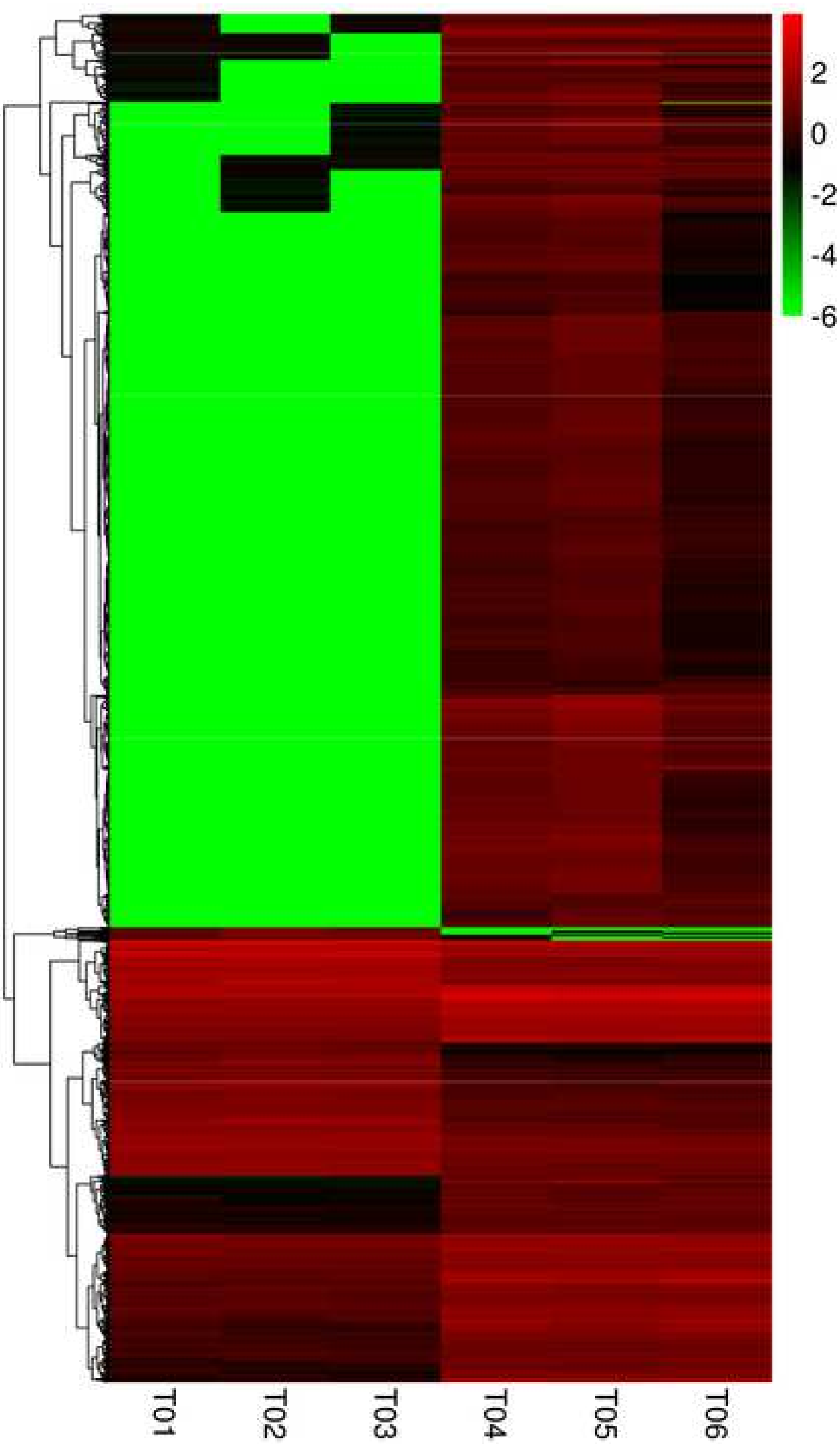

Figure8

Click here to download Figure Fig 8.tif

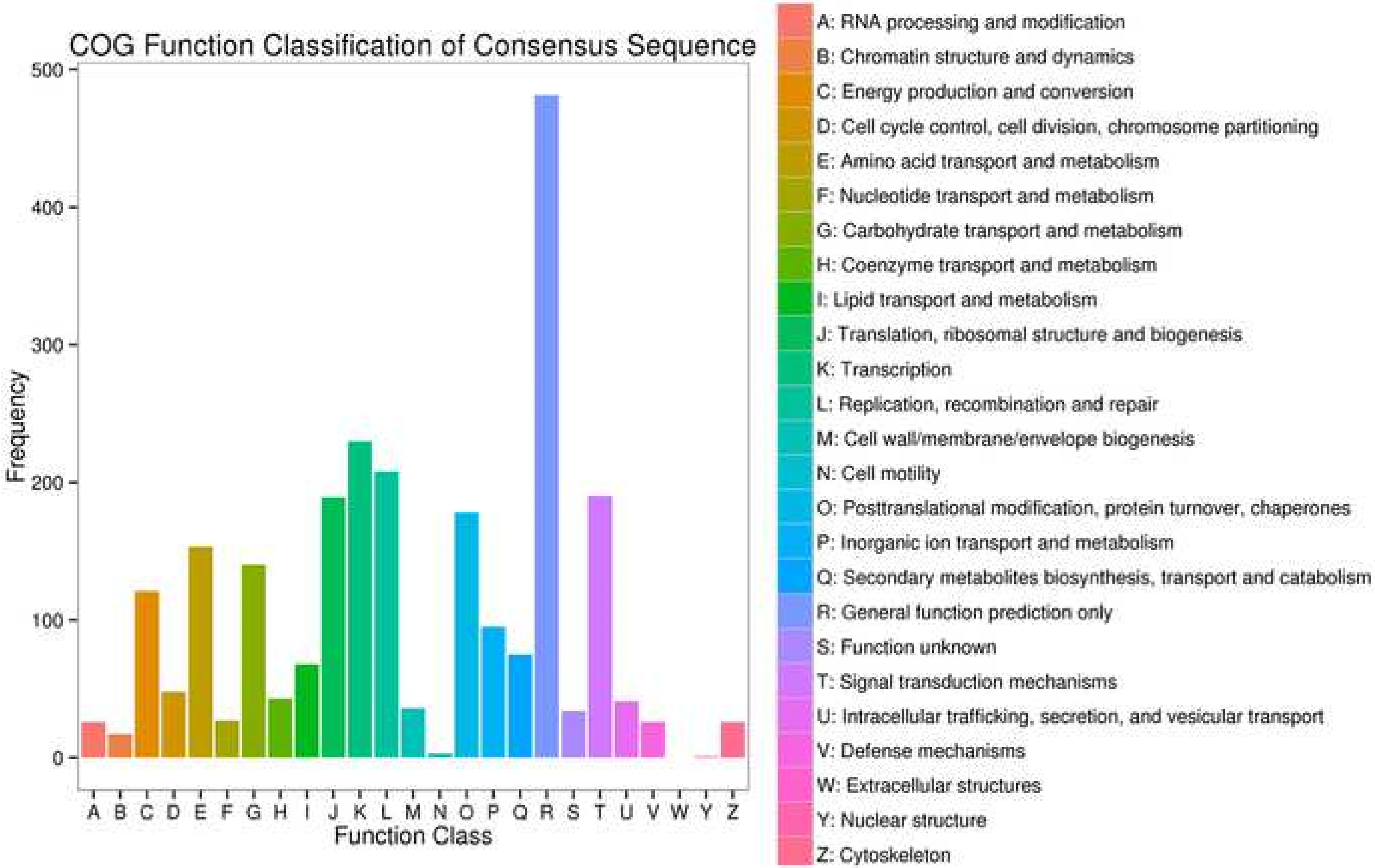

Figure9

Click here to download Figure Fig 9.tif

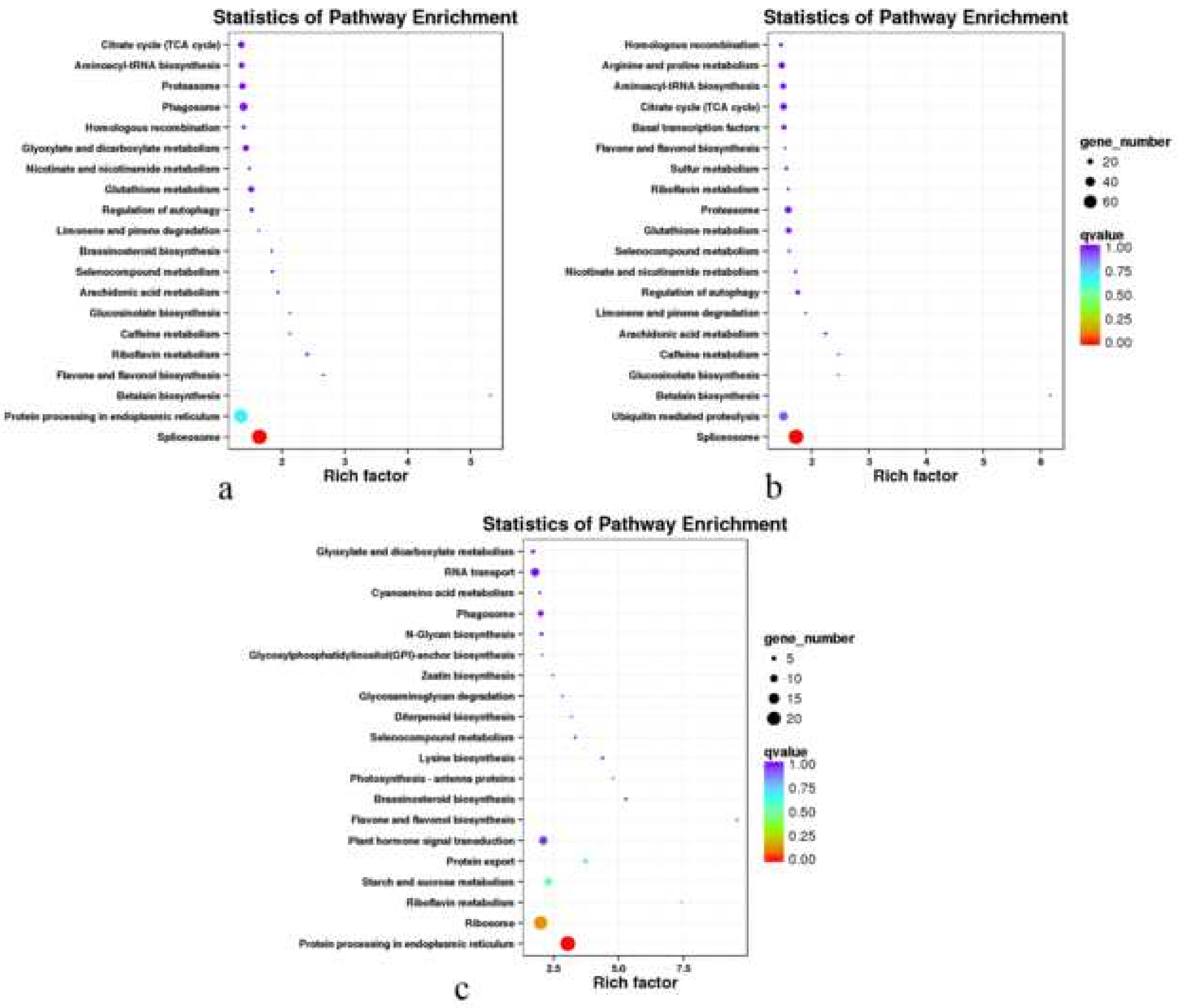

Figure10

Click here to download Figure Fig 10.tif

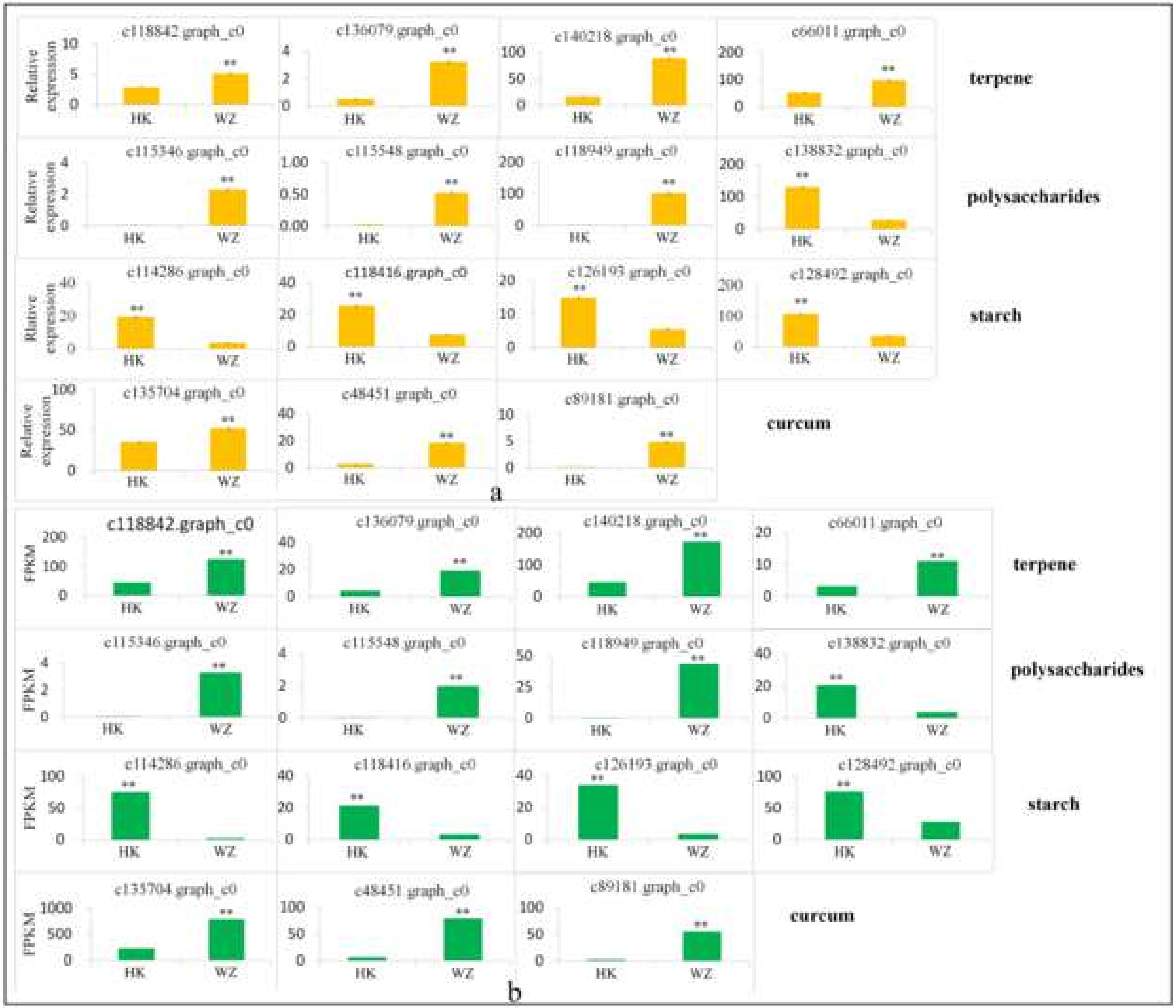

supporting Information

Click here to access/download
Supporting Information - Compressed/ZIP File Archive
Supporting information.rar
