# Supplementary material for: Transcriptome analysis of Curcuma wenyujin from Haikou and Wenzhou, and a comparison of the main constituents and related genes of Rhizoma Curcumae
Source: PLoS One. 2020 Nov 30;15(11):e0242776. doi: 10.1371/journal.pone.0242776 (PMC7703983; doi:10.1371/journal.pone.0242776)
Supplement: Attachment — Submitted filename: Response to Reviewers.docx [file pone.0242776.s021.docx]

Dear *Plos one* editor and reviewers,

We thank you for all of your comments and suggestions about ways to improve our manuscript, now retitled “Transcriptome analysis of *Curcuma wenyujin* from Haikou and Wenzhou and a comparison of the main constituents and related genes of Rhizoma Curcumae”. The following are our response to your questions and suggestions.

**Journal requirements:**

**1. When submitting your revision, we need you to address these additional requirements. Please ensure that your manuscript meets PLOS ONE's style requirements, including those for file naming. The PLOS ONE style templates can be found at http://www.journals.plos.org/plosone/s/file?id=wjVg/PLOSOne_formatting_sample_main_body.pdf and http://www.journals.plos.org/plosone/s/file?id=ba62/PLOSOne_formatting_sample_title_authors_affiliations.pdf**

Revision: Thanks for the suggestion, We have revised the manuscript to meet PLOS ONE's style requirements by consulting The PLOS ONE style templates.

**2. PLOS requires an ORCID iD for the corresponding author in Editorial Manager on papers submitted after December 6th, 2016. Please ensure that you have an ORCID iD and that it is validated in Editorial Manager. To do this, go to ‘Update my Information’ (in the upper left-hand corner of the main menu), and click on the Fetch/Validate link next to the ORCID field. This will take you to the ORCID site and allow you to create a new iD or authenticate a pre-existing iD in Editorial Manager. Please see the following video for instructions on linking an ORCID iD to your Editorial Manager account: https://www.youtube.com/watch?v=_xcclfuvtxQ**
Revision: Thanks for the suggestion, We have created a new ORCID：0000-0003-4317-0029

**3. Both reviewers pointed out deficiencies in description of methodological details, conditions and sample details and conditions. According to Publication Criterion #3 You are required to describe your methods and samples in sufficient detail to ensure replicability. This extends also to purchased products that may not be sufficiently described or characterized, or whose purchase locations were not provided.**

Revision: Thanks for the suggestion, I read it carefully and have made changes in the text. The purchased products was sufficiently described or characterized, purchase locations were provided in the paper. We have described methods and samples in sufficient detail to ensure replicability in the revised manuscript.

**4. You are required to state how the Illumina RNASeq was done (details and instrument) and submit the reads to NCBI SRA Archive (PloS ONE publication criterion #7 Data Availability (see also https://journals.plos.org/plosone/s/data-availability).**

Revision: Thanks for the suggestion, the data reads of Illumina sequencing have been deposited in the National Center for Biotechnology Information Sequence Read Archive (SRA accession: PRJNA598542; Temporary Submission ID: SUB6764411; SAMPLE: *Curcuma wenyujin* (SAMN13707351); Release date: 2020-01-08. https://www.ncbi.nlm.nih.gov/sra/PRJNA598542).

**Additional Editor Comments:**
**1. Is the manuscript technically sound, and do the data support the conclusions? The manuscript must describe a technically sound piece of scientific research with data that supports the conclusions. Experiments must have been conducted rigorously, with appropriate controls, replication, and sample sizes. The conclusions must be drawn appropriately based on the data presented. 
Reviewer #1: Partly
Reviewer #2: Yes**

Revision: Thanks for the suggestion, According to this suggestion “Experiments must have been conducted rigorously, with appropriate controls, replication, and sample sizes. The conclusions must be drawn appropriately based on the data presented. ” We have Modified this article based on detailed recommendations from experts in red highlight place.

**2. Is the manuscript presented in an intelligible fashion and written in standard English? PLOS ONE does not copyedit accepted manuscripts, so the language in submitted articles must be clear, correct, and unambiguous. Any typographical or grammatical errors should be corrected at revision, so please note any specific errors here.
Reviewer #1: Yes
Reviewer #2: No**

Revision: Thanks for the suggestion, This language polish has been corrected by the company(Editage) polish, to ensure clear, correct, and unambiguous.

**Review Comments to the Author**

**Reviewer** #**1”: The introduction part seems to be too lengthy – cab be shortened.**
Revision: Thanks for the suggestion, We have shortened the introduction in the revised manuscript.

**Reviewer #1: L.71: The terminology 'Species variety' is not botanically correct. Authors may use the term ‘genotype’ instead.**

Revision: Line 71: “Species variety” was replaced with “genotype”. Shown in line 69 in the revised manuscript.
**Reviewer #1: L.86: There is nothing called “lime soil”. Is it alkaline soil?**

Revision: Line 86: “lime soil” was replaced with “alkaline soil”. Shown in line83 in the revised manuscript.
**Reviewer #1: L.120-122: Give a suitable reference for this statement.**

Revision: Thanks for the suggestion, we have given a suitable reference for this statement in line 104 in the revised manuscript.

**Reviewer #1: L.146: What do you mean by treatment conditions?**

Revision: Thanks for the suggestion, L.146: “Plant materials and treatment condition**s? ”** was replaced with “Plant materials”. Shown in line 131in the revised manuscript.
**Reviewer #1: L.146-153: Authors silent on experimental conditions. Similar sampling and growing conditions are important for studying secondary metabolites and comparative transcriptomics. The environmental and weather conditions of the two provinces need to be given as supplementary table to understand the differences in secondary metabolites and related genes.**

Revision: Thank you for the recommends. The environmental and weather conditions of the two provinces was given in S1 Table (shown in L141 in the revised manuscript).
**Reviewer #1: L.154: Protocol for RNA isolation is not mentioned. Also specify the tissue from which RNA was isolated. In case of RNASeq, number of biological replicates sequences is not clear. Also, replicates need to be compared at sequence level and prove that they are not significantly different.**

Revision: L.154: “Protocol for RNA isolation and specify the tissue from which RNA was isolated ”is added in L143-145.Three biological replicates samples in sequences were mentioned, and were shown in line 145 in the revised manuscript. “replicates need to be compared at sequence level and prove that they are not significantly different” were mentioned in line 324-326 in the revised manuscript.
**Reviewer #1: L.209: Sufficient experimental details viz., temperature, pressure and slice thickness need to be given on boiling and drying of samples?**

Revision: L.209: “ Sufficient experimental details viz., temperature, pressure and slice thickness need to be given on boiling and drying of samples? ” were revised . Shown in line 205-207in the revised manuscript.
**Reviewer #1: L.234: What is the sample? rhizome or leaf, or at what stage?**

Revision: L.234: “What is the sample? rhizome or leaf, or at what stage? ” were revised with “*C. wenyujin* rhizomes ” . Shown in line 230 in the revised manuscript.
**Reviewer #1: L. 248: Is it a curcumin or curcuminoids**

Revision: Curcuminoids mainly cantain curcumin , demethoxycurcumin, bisdemethoxycurcumin and other curcumin substances, and in this paper, we analyzed a curcumin in rhizome of *Curcuma wenyujin*, so, "It is a curcumin in this paper" was confirmed, shown in line L244 in the revised manuscript.

**Reviewer #1: L.268: Polysaccharide and starch content methodology are too elaborate and need to shorten with suitable references.**

Revision: Thank you for the recommends. L.268: “Polysaccharide and starch content methodology are too elaborate and need to shorten with suitable references” was revised and shorten with suitable references[53-54]. Shown in line L264-279 in the revised manuscript.

**Reviewer #1: In many literatures, the C. weeyujin is referred as synonymous to C. aromatica. In that case can authors compare the present transcritomes with already available C. aromatica transcriptome to draw the meaningful conclusions. Hope above suggestions help authors to improve the manuscript.**
Revision: Thank you for the recommends. In discussions, We cited the literature and discussed and compared *C. wenyujin* between HK and WZ areas. Shown in line L486-489 in the revised manuscript.

**Reviewer #2: Language correction is essential before acceptance. Method used for RNA isolation is not mentioned (what mentioned as RNAse free DNAse, Takara; is just an enzyme and is not the method used for RNA extraction).**
Revision: This language polish has been corrected by the company (Editage) polish, and RNA extraction methods have been mentioned in the article L43-145.
**Reviewer #2: Line no. 38, 39: better replace ‘was’ with were.**

Revision: Line 38, 39: “was” was replaced with “were”. Shown in line 39 in the revised manuscript.

**Reviewer #2: Species name should always be in small letters, throughout the manuscript its written as Curcuma Wenyujin, few line numbers are mentioned ( Line no.119, 134, 148, 342, 350, 347, 355, 506, 509, 522, 528, 538, 541, 544, 546, 549, 558, 562, 563, 574, 582, 583, 594, 609, 611, 625, 627, 644, 650).**

Revision: Line (119, 134, 148, 342, 350, 347, 355, 506, 509, 522, 528, 538, 541, 544, 546, 549, 558, 562, 563, 574, 582, 583, 594, 609, 611, 625, 627, 644, 650) “Species name should always be in small letters, throughout the manuscript its written as *Curcuma Wenyujin* ” were revised. Shown in Line (100, 111, 134, 289, 295, 297, 302, 457, 461, 474, 480, 491, 493, 497, 498, 501, 510, 512, 513, 515, 524, 532, 533, 544, 559, 561, 574, 577, 594) in the revised manuscript.
**Reviewer #2: Make sure that Curcuma wenyujin is in italics (not followed).**

Revision: “*Curcuma wenyujin* is in italics ” were revised . Shown in the revised manuscript.
**Reviewer #2: Line no. 265: Rephrase the sentence in a better conveyable form.**

Revision: Line no. 265“Rephrase the sentence in a better conveyable form”were revised . Shown in line 260-265 in the revised manuscript.
**Reviewer #2: Line no. 297: spelling correction; ‘using’ is written as uing.**

Revision: Line no. 297 “uing” was replaced with “using”. Shown in line 267 in the revised manuscript.
**Reviewer #2: Line no. 329: better replace ‘was’ with were.**

Revision: Line no. 329 was revised. Shown in line 274-279 in the revised manuscript.
**Reviewer #2: Line no. 561 is contradictory to Line no. 564 (about polysaccharide).**

Revision: Line no561-564 “561 is contradictory to Line no. 564 (about polysaccharide) in Line no.561-564” were revised . Shown in line 511-515 in the revised manuscript.

**Other revisions:**

**1. Line 469** “**15 of the transcriptomes of differentially expressed genes- How did you select these genes? What was the criteria followed to shortlist 15 genes?** ”

Revision: These 15 genes are genes related to terpenoids, curcumin, polysaccharides, and starch metabolism pathways of *Rhizoma Curcuma*, and the selected genes are expressed in both samples. The differential expression of genes in the two regions is high, and the p value and FDR value are within the range. These genes are designed with appropriate primers and quantified.

**2. Line 376**“**A total of six samples-what are they?** ”

Revision: Thank you for the recommends. they are HK1, HK2, HK3, WZ1, WZ2, WZ3. Shown in line 326-327 in the revised manuscript.

Best regards,

Lulilan,
