# Supplementary material for: Transcriptome analysis of Curcuma wenyujin from Haikou and Wenzhou, and a comparison of the main constituents and related genes of Rhizoma Curcumae
Source: PLoS One. 2020 Nov 30;15(11):e0242776. doi: 10.1371/journal.pone.0242776 (PMC7703983; doi:10.1371/journal.pone.0242776)
Supplement: Attachment — Submitted filename: Response to Reviewers.docx [file pone.0242776.s022.docx]

Dear *Plos one* editor and reviewers,

We thank you for all of your comments and suggestions about ways to improve our manuscript, and give us a second chance to revise the paper, now retitled “Transcriptome analysis of *Curcuma wenyujin* from Haikou and Wenzhou, and a comparison of the main constituents and related genes of Rhizoma Curcumae”. The following are our response to your questions and suggestions.

**Journal requirements:**
While some of the major issuses raised by reviewers in the previous round were addressed more work is required improve descriptions of methods and language to meet publicaiton criteria #3 and #5. Especially, add parameters and thresholds to methods applied, explain rational for chosing a particular statistical test (here univariate ANOVA), and most importantly revisit and revise the DEG analysis part to ensure enrichment results are statically correctly supported. In additon your are required to improve lablelling and quality of figures.

**Revision:** Thank you very much for the suggestion, and We have accepted the expert's suggestion that we improve the description of the method and language by polishing the company to meet the #3 and #5 public standards. In particular, Why are parameters and thresholds added to the applied method to explain the rationality of choosing a specific statistical test (here, univariate ANOVA), Curcuma rhizomes from different species, and they have only two groups, We had applied univariate ANOVA instead of t-test or U-test. Seen in lines 282 in the revised. and the DEG analysis part and enrichment results have been modified and explained. In addition, we also made adjustments to meaningful tags and improved image quality. We have Modified this article based on detailed recommendations from experts in red highlight place.

**Comments to the Author**
1. If the authors have adequately addressed your comments raised in a previous round of review and you feel that this manuscript is now acceptable for publication, you may indicate that here to bypass the “Comments to the Author” section, enter your conflict of interest statement in the “Confidential to Editor” section, and submit your "Accept" recommendation.
Reviewer #2: All comments have been addressed
Reviewer #3: (No Response)
2. Is the manuscript technically sound, and do the data support the conclusions?
The manuscript must describe a technically sound piece of scientific research with data that supports the conclusions. Experiments must have been conducted rigorously, with appropriate controls, replication, and sample sizes. The conclusions must be drawn appropriately based on the data presented.
Reviewer #2: Yes
Reviewer #3: Partly

Revision: Thanks for the suggestion, According to this suggestion “Experiments must have been conducted rigorously, with appropriate controls, replication, and sample sizes. The conclusions must be drawn appropriately based on the data presented. ” We have Modified this article based on detailed recommendations from experts in red highlight place.
3. Has the statistical analysis been performed appropriately and rigorously?
Reviewer #2: Yes
Reviewer #3: No

Revision: Thanks for the suggestion, According to this suggestion “the statistical analysis been performed appropriately and rigorously. ” and according to this suggestion We had applied univariate ANOVA instead of t-test or U-test. Seen in lines 282 in the revised manuscript. We have Modified this article based on detailed recommendations from experts in red highlight place.
4. Have the authors made all data underlying the findings in their manuscript fully available?
The [PLOS Data policy](http://www.plosone.org/static/policies.action) requires authors to make all data underlying the findings described in their manuscript fully available without restriction, with rare exception (please refer to the Data Availability Statement in the manuscript PDF file). The data should be provided as part of the manuscript or its supporting information, or deposited to a public repository. For example, in addition to summary statistics, the data points behind means, medians and variance measures should be available. If there are restrictions on publicly sharing data—e.g. participant privacy or use of data from a third party—those must be specified.
Reviewer #2: Yes
Reviewer #3: Yes
5. Is the manuscript presented in an intelligible fashion and written in standard English?
PLOS ONE does not copyedit accepted manuscripts, so the language in submitted articles must be clear, correct, and unambiguous. Any typographical or grammatical errors should be corrected at revision, so please note any specific errors here.
Reviewer #2: Yes
Reviewer #3: No

Revision: Thanks for the suggestion, This language polish has been corrected by the company(Editage) polish, to ensure clear, correct, and unambiguous. Seen in red highlight place in the revised manuscript.

6. Review Comments to the Author
Reviewer #2: Authors incorporated the suggestions into their manuscript and as a result the MS quality has been improved. The revised MS can be accepted as it is.

Answer: Thank you for your comments
Reviewer #3:

The authors analyzed C. wenyujin rhizomes in two different locations and showed that the difference in metabolome and transcriptome were consistent. The conclusion sounds OK but there are so many points to be improved in figures, statistical analysis and sentences. The comparison along the metabolic pathway, and their conclusion would be OK. However, the distribution of transcriptome should be evaluated and normalized before detail statistical analysis such as DEG. Also, the detail about clustering analysis and enrichment analysis are not clear to evaluate them.

Revision: Thank you for your comments, the distribution of transcriptome was evaluated and normalized before detail statistical analysis such as DEG and unigenes in S1-Fig, S4-S7 Table. Also, the detail about clustering analysis and enrichment analysis are evaluate them in S9, S10 Table and S1 File. In-depth research in the future, we will further analyze.
All of the figures were looks very low in resolution (converted from compressed JPEG?) and most of the labels are difficult to read, even I downloaded each tiff file.Use vector format images or, at least, more high resolution and low compression images.

Revision: Thank you for your comments, all of the figures were adjusted by using more high resolution and low compression images.
It is strongly recommended to submit the whole manuscript to an English editorial service.

Revision: Thank you for your comments, We had submited the whole manuscript to an English editorial service again (Editage) . Seen in red highlight place.

There are detail comments below.
L85. ~ indicating that the oil content of Curcuma rhizomes can be affected by factors such as soil quality and climate. Add some reference if there are some preceding related studies.

Revision: Thank you for your comments. We have added a reference about preceding related studies, However, now, There is still not much research on related studies about the soil quality and climate, in lines L84-88 in the revised manuscript.

L192: It would be specify the name of the test and parameters since "DESeq" is a name of a function in the R package rather than a name of a method.
Revision: Thank you for your comments. "DESeq" is revised for a name of a function in the R package. The P value results were regulated using the Benjamin and Hochberg method (1995) for controlling the false discovery rate (FDR), and genes at a P value < 0.05 were classified as differentially expressed genes (DEGs), in lines L198-202 in the revised manuscript.
L282 : Univariate analysis of variance was applied to determine the significance of the results among different treatments. The target of the analysis unclear. Does it means "different species", and the variables are compound components? Since they have only two groups, are there any rational reason to apply univariate ANOVA instead of t-test or U-test?

Revision: Thank you for your comments. Curcuma rhizomes from different species, and they have only two groups, We had applied univariate ANOVA instead of t-test or U-test. Seen in lines 288 in the revised manuscript.
L:327 41.74 Gb, If it means giga "byte", show the amount in giga "base pair" instead.

Revision: Thank you for your comments, it means giga "byte", 41.74 Gb was revised for 41.74 Gbp (base pair) in lines 335 in the revised manuscript.
L: 328 Q30 percentages, It should be more concretely and use general term.
For example, "Number of samples which Phred quality score is higher than 30."

Revision: Thank you for your comments, Q30 percentages was revised for general term that number of samples which Phred quality score is higher than 30) in lines 336-337 in the revised manuscript.
L 340: 1.00e-05, It would be better to use scientific format (superscripts) instead of computational format.

Revision: Thank you for your comments. It has been used scientific format (superscripts)( 1.00-5) instead of computational format in lines 342 in the revised manuscript.
L 340: In the DEG analysis the number of up regulated genes were much larger than that of down regulated. The authors should concern about experimental bias between samples or consider to apply some compensation such as quantile normalization.
Revision: Thank you for your comments. Yes, you are right about that “experimental bias between samples or consider to apply some compensation such as quantile normalization”. In the DEG analysis the number of up /down regulated genes, logarithm quantile normalization (Log2FC（WZ/HK）) were seen in S8 Table .

Fig 4 shows the ratio of DEG unigenes in each ontology is highly correlated with that of all annotated unigenes.It seems that DEG are not correlate with their functions but almost randomly sampled from the all unigenes, so that the significance of enrichment analysis is rather doughtful.

Revision: Thank you for your comments. Fig4 showed Gene ontology (GO) histogram of classification in annotated unigenes WZ (Wenzhou) versus HK (Haikou). The functions of the unique sequences extracted from C. wenyujin were classified based on the three GO terms: cellular component (CC), biological process (BP), and molecular function (MF), The DEGs were mainly assigned to ‘cell, go: 0005623’, ‘cell part, go: 0044464’, ‘organelle, go: 0043226’, ‘membrane, go: 0016020’,‘cellular process, go: 0009987’, ‘metabolic process, go: 0008152’, ‘single-organism process, go: 0044699’, ‘binding, go: 0005488’, and ‘catalytic activity, go: 0003824’ (Fig 4). and We had some KEEG and GO enrichment analysis seen in lines 405-418 and 446-458 in the revised manuscript and related enrichment information was seen in S9, S10 Table and S1 File. We will conduct more in-depth research in the future on the significance of enrichment analysis.
Fig. 7: Make clear the number of genes shown in this figure, is it 3720? If it is so make clear how and why these genes were chosen. It is not clear what means "up/down regulated" in this figure, I mean compared with what values.And the labels in the columns should be replaced by their real labels (T01 = HK1, T02 = HK2, T03 = HK3, T04 = WZ1, T05 = 393 WZ2, T06 = WZ3).

Revision: Thank you for your comments. Make clear the number of genes shown in this figure is 4620. These genes were chosen because of a significant difference in these genes between WZ and HK . the values of up/down regulated" in this figure were those of WZ compared HK. And the labels in the columns have been replaced by their real labels (HK1, HK2, HK3, WZ1, WZ2, WZ3) in Fig 7 in the revised manuscript.
L409: A comparison of DEGs in 26 COG classifications between HK and WZ is shown in Fig 8. It is not clear that the numbers of DEG in this figure means up regulated or down regulated. And for enrichment analysis, it is also important to show the number of annotated genes in each category and the ratio of DEG to the annotated genes.
Revision: Thank you for your comments, We mean that 26 COG functional classifications of consensus sequence of DEGs between HK and WZ are shown in Fig 8, not a comparison of DEGs in 26 COG classifications between HK and WZ is shown in Fig 8. We have revised it seen in Line 409. We have showed the number of annotated genes in main eight categories and the ratio of DEG to the annotated genes, seen in lines 424-432in the revised manuscript.
L 454:It seems meaningless to show the sequence id such like c118842.grraph-co, show gene name or protein name instead. It would be much better to show them in the pathway map also.

Revision: Thank you for your comments, We have showed gene name or protein name instead of the sequence id such like c118842.grraph-co seen in lines 475-478 in the revised manuscript.
Fig. 10Arrange the plots for FPKM and rt-PCR side by side when the comparison about the same gene is important.

Revision: Thank you for your comments, We have arranged the plots for FPKM (DGE) and RT-PCR side by side when the comparison, seen in Fig. 10 in the revised manuscript.
7. PLOS authors have the option to publish the peer review history of their article ([what does this mean?](https://journals.plos.org/plosone/s/editorial-and-peer-review-process)). If published, this will include your full peer review and any attached files.If you choose “no”, your identity will remain anonymous but your review may still be made public.

Answer: Thank you, We choose “no”.
